# Fractal photonic anomalous Floquet topological insulators to generate multiple quantum chiral edge states

**DOI:** 10.1038/s41377-023-01307-y

**Published:** 2023-11-02

**Authors:** Meng Li, Chu Li, Linyu Yan, Qiang Li, Qihuang Gong, Yan Li

**Affiliations:** 1 State Key Laboratory for Artificial Microstructure and Mesoscopic Physics, School of Physics, Peking University, Beijing, 100871 China; 2 Frontiers Science Center for Nano-Optoelectronics, Peking University, Beijing, 100871 China; 3 Collaborative Innovation Center of Extreme Optics, Shanxi University, Taiyuan, Shanxi 030006 China; 4 Hefei National Laboratory, Hefei, 230088 China; 5 Peking University Yangtze Delta Institute of Optoelectronics, Nantong, 226010 China

**Keywords:** Quantum optics, Single photons and quantum effects, Integrated optics

## Abstract

Anomalous Floquet topological insulators with vanishing Chern numbers but supporting chiral edge modes are attracting more and more attention. Since the existing anomalous Floquet topological insulators usually support only one kind of chiral edge mode even at a large lattice size, they are unscalable and unapplicable for multistate topological quantum systems. Recently, fractal topological insulators with self-similarity have been explored to support more nontrivial modes. Here, we demonstrate the first experimental realization of fractal photonic anomalous Floquet topological insulators based on dual Sierpinski carpet consisting of directional couplers using the femtosecond laser direct writing. The fabricated lattices support much more kinds of chiral edge states with fewer waveguides and enable perfect hopping of quantum states with near unit transfer efficiency. Instead of zero-dimensional bound modes for quantum state transport in previous laser direct-written topological insulators, we generate multiple propagating single-photon chiral edge states in the fractal lattice and observe high-visibility quantum interferences. These suggest the successful realization of highly indistinguishable single-photon chiral edge states, which can be applied in various quantum operations. This work provides the potential for enhancing the multi-fold manipulation of quantum states, enlarging the encodable quantum information capacity in a single lattice via high-dimensional encoding and many other fractal applications.

## Abstract (web summary)

We demonstrate the experimental realization of fractal photonic anomalous Floquet topological insulators for generating multiple single-photon chiral edge states in a single lattice fabricated by the femtosecond laser direct writing.

## Graphical abstract

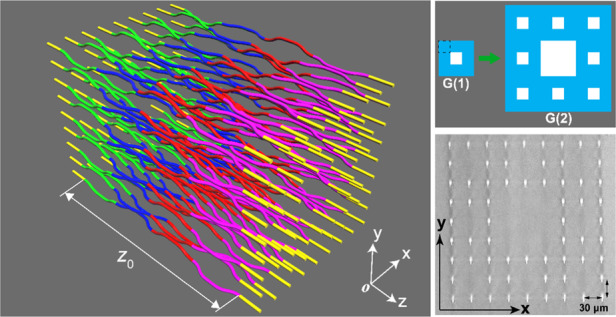

## Introduction

Anomalous Floquet topological insulator (AFTI) has nonzero winding numbers to support topological edge modes^1^, though its standard topological invariants like Chern numbers are zero. Quantum simulation of AFTIs has been experimentally explored in various physical systems, such as photonic^2–5^, acoustic^6–8^, cold atomic^9,10^ and so on. The photonic lattice fabricated by the femtosecond laser direct writing (FLDW) is a promising platform for quantum simulation of various topological insulators (TIs), because the FLDW offers flexible design of true three-dimensional (3D) waveguide structures and precise control of each coupling between waveguides^11–14^. In laser direct-written photonic AFTIs, selective coupling of adjacent waveguides in a cycle is explicitly defined by the discrete periodically driving protocol. By modulating the driving protocol, photonic AFTIs exhibit abundant functions. Selecting specific driving protocols, chiral edge modes co-exist with dispensionless bulk modes^2,3^. By combining effective fermionic time-reversal symmetry, photonic version of an electronic TI supporting counter-propagating chiral edge modes was realized^4^. Through nonlinear optical Kerr effect, the formation of solitons was observed in photonic AFTIs^15,16^ and the trivial AFTI was changed into a topologically nontrivial regime when the input optical power was increased above a certain threshold with the modified driving protocol^17^. In addition, the photonic AFTI with perfect hopping protocol has advantages of robust topological edge transport of quantum states with the highest transfer efficiency (~100%) among all TIs^2,18^.

Although photonic AFTIs have been widely investigated, most of them usually support only one kind of chiral edge mode even at a large lattice size, exhibiting just one chirality and only propagating along the outer boundaries of lattices, which are unscalable and unapplicable for multistate topological quantum systems. Recently, the emerging fractal TIs generated by selectively removing lattice sites from the normal TIs have attracted much attention for their self-similarity and non-integer dimension. In fractal photonic Floquet topological insulators constructed by helical waveguides^18,19^, the original outer edge states and the generated inner edge states are both topological nontrivial, which goes beyond the confines of the bulk-boundary correspondence. In fractal high-order topological insulators based on static two-dimensional (2D) Su–Schrieffer–Heeger (SSH) or Benalcazar–Bernevig–Hughes (BBH) model^20,21^, there are much richer topological states than conventional 2D systems, such as outer and inner corner states. Moreover, some fractal-like photonic lattices were proposed to explore the behavior of multiple localized states emerging from singular and nonsingular flat bands^22,23^. With the growth of fractal generations, the advantage of supporting more topological nontrivial modes with fewer bulk sites using a single lattice becomes more significant.

Though the fractal photonic AFTI was touched upon in the theoretical simulation^18^, it has not been experimentally realized, yet. In 2018, the group of Jin demonstrated the quantum transport in fractal networks constructed by straight waveguide arrays^24^, where they used the coherent light beam instead of single photons to perform continuous-time quantum walks. So far, all experimental works on quantum state transport in laser direct-written photonic TIs have been limited to the zero-dimensional topological bound states, such as topological boundary states in one-dimensional (1D) SSH models or Harper models^25–29^, and corner states in 2D higher-order TIs^30,31^, exhibiting no topological edge transport properties.

Here, we demonstrate the first experimental realization of fractal photonic AFTIs based on dual Sierpinski carpet (DSC) using FLDW and high-visibility (above 90%) quantum interferences of multiple single-photon chiral edge states that dynamically transport along various boundaries of the fractal lattice. The fabricated fractal photonic AFTIs with fewer waveguides not only support much more chiral (unidirectional) edge modes, but also enable perfect hopping of topological quantum states. The successful generation and control of indistinguishable single-photon chiral edge states shows potentials in generating topologically protected entangled states or performing quantum logical operations. The emerged multiple inner edge modes due to fractal’s self-similarity enlarges the encodable quantum information capacity carried by a single lattice via high-dimensional encoding for quantum fractal pattern states. This work lays the foundation for realizing scalable topological photonic quantum computing, constructing topologically protected high-dimensional multistate quantum information processing systems, and can extend to many other fractal structure applications.

## Results

**Fig. 1:**
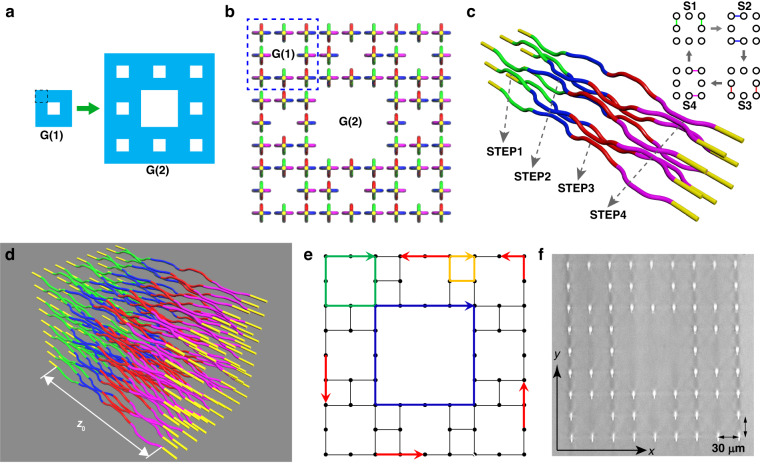
Photonic implementation of a fractal anomalous Floquet topological insulator (AFTI). **a** The first and second generation of Sierpinski carpet (G(1) and G(2)). The black dashed square represents a lattice site, so the fractal structure becomes the dual Sierpinski carpet (DSC). **b** The pattern of lattice sites with the fractal structure of the DSC. The part in the blue dashed square represents G(1), and the whole is the G(2). **c** A schematic sketch of eight lattice sites (G(1)) to show how the perfect hopping driving protocol of the four-step model (inset) in one period is implemented using 3D waveguides: in each step, only one kind of coupling is switched on when two waveguides approach close to form a DC and the other three are off, and the theoretical transmittivity of each DC is set as 100%. **d** A schematic sketch of the fabricated sample with 64 lattice sites (G(2)) in one period *z*_0_ = 24.6 mm. **e** In the case where all coupling strengths are set equal, $\varLambda =\kappa {z}_{0}/4=\pi /2$, chiral outer (red) and inner (blue, green) edge modes arise, while the bulk (yellow) mode is localized. Inner edge modes IE_A_ and IE_B_ are distributed at the inner peripheries of the central hole of G(2) lattice and the holes in the sub squares represented by G(1) lattice, respectively. **f** Micrograph of the facet of the fractal photonic lattice fabricated by FLDW. The initial spacing of adjacent sites is set as 30 μm to prevent them from undesired coupling, so the transverse size is 240 μm × 240 μm

The lattice cross-section of most AFTIs is square, so we select the square Sierpinski carpet, one of the most famous fractal structures, to construct fractal photonic AFTIs. Its basic iterative process is shown in Fig. 1a. There are two ways to construct the fractal lattice^24^. Taking the first-generation G(1) for example, one way is to replace the 4 corners of small squares in blue (one of which is enclosed by the black dashed line) with sites, requiring 4 × 8 = 32 sites; and the other is to replace the small square in blue itself with a site, requiring 1 × 8 = 8 sites. Both lattices have the same Hausdorff dimension of *d*_f_ = ln(8)/ln(3) ≈ 1.89, but the latter one requiring fewer lattice sites is easier to fabricate. We arrange the fractal lattice in DSC structure at the second-generation G(2) with 64 sites (Fig. 1b), and fabricate the lattice inside glass by FLDW (Fig. 1f). The one-period waveguide structure of G(1) is constructed by a series of discrete horizontal and vertical directional couplers (DCs), arranged at the 4-step perfect hopping driving protocol (Fig. 1c) to obtain the highest transfer efficiency and preserve chiral edge modes. The individual couplings are guaranteed by the specially designed 3D waveguide structure based on DCs, quite different from the previous fractal lattices constructed by identical straight waveguides or helical waveguides^18,24^. The one-period waveguide structure of G(2) with 88 DCs is shown in Fig. 1d, which is already able to fully demonstrate the fundamental features of the fractal AFTI. In Fig. 1e, there exist four kinds of modes according to their transport behaviors, which not only perseveres the anticlockwise outer edge modes (red arrows) and part of dispersionless bulk modes (yellow arrows), but also supports two clockwise inner edge modes IE_A_ (blue arrows) and IE_B_ (green arrows), enriching the type of chiral edge modes in a single lattice.

The Hamiltonian of the system is *z*-dependent, simulating the time-dependence. Considering the periodicity in the propagating direction, the Floquet state *ψ* meets^2,3,5^:

1 $$\psi (t)=\varphi (t){e}^{-i\varepsilon t}\,(\varphi (t+T)=\varphi (t))$$

where *ε* is the quaisenergy, *T* is the period of the function *φ*. The temporal evolution of the Floquet state can be described as:

2 $$\psi (t)=P{e}^{-i{\int }_{0}^{t}H(\tau )d\tau }\psi (0)$$

with $\psi (T)={e}^{-i\varepsilon T}\psi (0)$. *P* is the time-ordering operator. Considering the 4-step driving protocol, the time evolutionary operator ${\hat{U}}(t)=P{e}^{-i{\int }_{0}^{t}H(\tau )d\tau }$ in a spatial period ${z}_{0}$ is:

3 $${\hat{U}}({z}_{0})={e}^{-i{\hat{H}}_{4}{z}_{0}/4}{e}^{-i{\hat{H}}_{3}{z}_{0}/4}{e}^{-i{\hat{H}}_{2}{z}_{0}/4}{e}^{-i{\hat{H}}_{1}{z}_{0}/4}$$

where the Hamiltonian ${\hat{H}}_{i}$ (*i* = 1, 2, 3 and 4) in each spatial interval ($(i-1)\,{z}_{0}/4\le z\le i\,{z}_{0}/4$) represents main features of the fractal lattice. For each step, the coupling coefficient is ${\kappa }_{i}$, and the coupling strength is set as ${\varLambda }_{i}={\kappa }_{i}{z}_{0}/4$=$\pi /2$ ($i$ = 1, 2, 3 and 4). The eigenvalue of the transform matrix ${\hat{U}}({z}_{0})$ is ${e}^{-i\phi }$, so the quasienergy is $\varepsilon =\phi /T$, whose range is $[-\pi /T,\,\pi /T]$, and *T* can be replaced by the spatial period ${z}_{0}$.

**Fig. 2:**
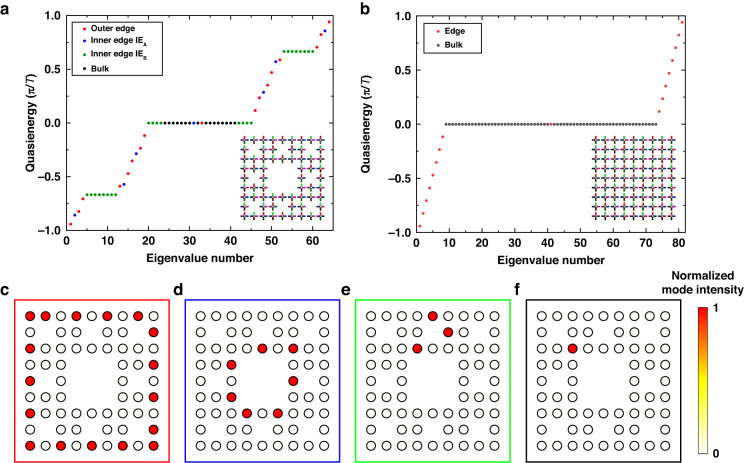
Comparison of the fractal and the normal photonic anomalous Floquet topological insulator and numerically calculated quasienergy spectrums. **a** Quasienergy spectrum for the 64-site fractal photonic lattice, where the black dots represent the bulk modes, and the red, blue, and green dots represent outer edge modes, inner edge modes IE_A_ and IE_B_, respectively. **b** Quasienergy spectrum for the 81-site normal photonic lattice, where the black and the red dots represent the bulk and the edge modes. **c**–**f** The field intensities of outer edge modes, inner edge modes IE_A_ and one of inner edge modes IE_B_ and one of bulk modes. More details about degenerate inner edge IE_B_ modes and bulk modes are shown in the Supplementary Information

By selective removal of certain lattice sites, the fractal photonic AFTI with fewer waveguides supports much more chiral edge modes than the normal one, as shown in the comparison of quasienergy spectrums (Fig. 2a, b). The normal AFTI with 81 sites only has two kinds of modes: 17 chiral edge modes and 64 dispersionless bulk modes. By contrast, the fractal AFTI with 64 sites supports four kinds of modes: 17 outer edge modes, 7 inner edge modes IE_A_, 24 inner edge modes IE_B_ and 16 bulk modes. Thus, the number of chiral edge modes carried by a single lattice increases greatly to 48. The fractal lattice is originated from the normal one, so both have the same field intensities of outer edge modes (Fig. 2c) and part of bulk modes (Fig. 2f). The emergence of inner edge modes due to fractal’s self-similarity is the significant difference. As shown in Fig. 2d, the inner edge mode IE_A_ is analogous to the outer edge mode, but its energy flows at the inner boundary of the central hole and has the opposite chirality (clockwise) to the anticlockwise outer edge mode. Inner edge modes IE_B_ are eightfold degenerate, related to the eight units of G(1) (sub squares), as shown in Fig. 2e. The degenerate inner edge modes distributed at different spatial positions are suitable for high-dimensional spatial encoding, and the opposite chirality of outer and inner edge modes can be used for chiral encoding. One fascinating property about fractals is that each constituent exhibits the same character as the whole, especially for higher generations. The pattern formed by the total distribution of modes in the G(2) lattice can be regard as an information carrier via high-dimensional encoding, thus the G(3) lattice including eight G(2) patterns carries a larger amount of information. What’s more, the G(3) lattice supports much richer controllable chiral edge modes with nontrivial distributions, displayed in the Supplementary Information.

**Fig. 3:**
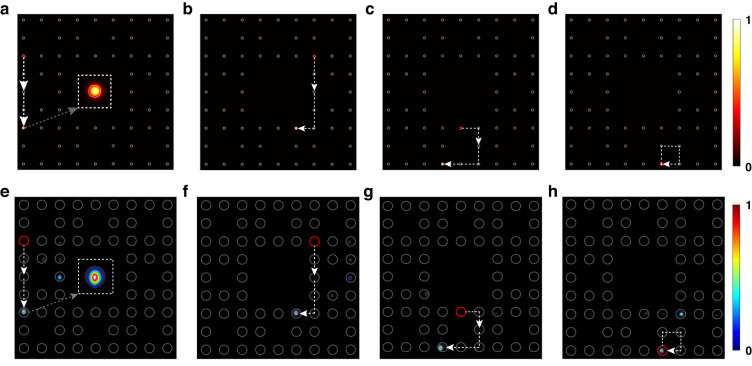
Topological mode transport in the dual Sierpinski carpet lattice after two full periods by single-site excitation. The light is injected into the lattice site indicated by the red circle. Simulation of the mode transport by beam-propagation-method (BPM): **a** Anticlockwise transport of the outer edge mode; **b** Clockwise transport of the inner edge mode IE_A_; **c** Clockwise transport of the inner edge mode IE_B_; **d** Localization of bulk mode. White circles represent cross sections of waveguides in simulation. Experimentally observed output intensity distribution: **e** outer edge mode; **f** inner edge mode IE_A_; **g** inner edge mode IE_B_; **h** bulk mode. The experimental results match well with the theory. The insets in **a** and **e** are the enlarged view of the waveguide modes. The thin white dash arrows describe the energy flow in the lattice. Gray circles just represent positions of fabricated waveguides instead of cross sections

We fabricate both the one-period and two-period fractal AFTIs in the 70-mm-long borosilicate glass (Eagle2000, Corning) using FLDW, and details can be found in the “Method” section. To excite chiral edge modes, bulk modes and to match the property of single photons, we inject the laser beam at 808 nm with vertical polarization into single lattice sites and measure the dynamical evolution of photons transporting in the lattice. The numerical simulation and the experimental results are shown in Fig. 3a–h, respectively. The observed topological edge transport behavior of outer edge modes and inner edge modes in two periods exhibits scattering-free propagating along corresponding boundaries and turning all corners. The dispersionless bulk modes are localized to the injection sites after one and two periods. In theoretical simulation, photons can completely transfer from sites to sites under designed driving protocol. The experimental results match quite well with the simulation, even though a fraction of photons leaks to other undesired sites due to the imperfection of fabrication and incomplete transfer at DCs. In addition, photonic AFTIs in the triangle Sierpinski gasket using the three-step model are demonstrated in the Supplementary Information.

**Fig. 4:**
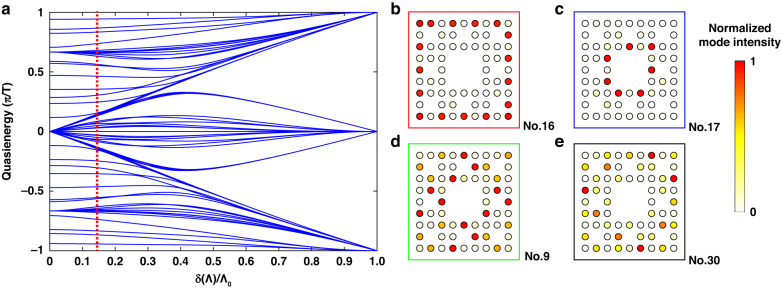
Topological robustness of chiral edge modes in fractal photonic AFTIs. **a** Quasienergy spectrum for the fractal lattice with the increasing deviation of coupling strength $\delta (\varLambda )/{\varLambda }_{0}$, where ${\varLambda }_{0}=\pi /2$. The red dashed line marks the deviation of 15%. **b**–**e** Field intensities of four typical modes when the deviation strength is 15%: **b** the outer edge mode (No. 16). **c** the inner edge mode IE_A_ (No. 17). **d** The inner edge mode IE_B_ (No. 9). It shows the sum distribution of 8 single inner edge modes IE_B_. **e** The bulk mode (No. 30)

To verify whether chiral edge modes are topologically protected or not, their topological invariants should be provided, but it is difficult to quantify in this aperiodic fractal AFTI system for the invalidation of existing methods (Discussions can be seen in Supplementary Information). It is believed that a better one may be found in the future with the development of fractal topological theory. Therefore, we just study the topologically protected dynamics of three chiral edge modes^32^, requiring their unidirectional edge transports should be robust against disorders and defects. The changes of refractive index for waveguides induced inside glass decrease with the increasing writing depths, embodied in the difference of waveguide cross sections at each layer (Fig. 1f). This results in the (diagonal) disorder of on-site energy, but it is insignificant as confirmed in the ref. ^3^ and by our experimental results. The off-diagonal disorder is corresponding to the slight fluctuation of coupling strengths due to the imperfection of fabrication. To determine the influence of deviations, quasienergy spectrums for the increasing deviation strength $\delta (\varLambda )/{\varLambda }_{0}$ (${\varLambda }_{0}$ = *π*/2) are displayed in Fig. 4a. With the increasing deviation, the dispersion of bulk modes becomes apparent, but the quasienergy of partial chiral edge modes remain nearly unchanged even the deviation strength reaches 15% and the transmissivity decreases from 100% to 95%.

Contrasted with Fig. 2c, d, the distributions of field intensities of outer edge mode (Fig. 4b) and inner edge mode IE_A_ (Fig. 4c) change slightly. The distribution of field intensities of inner edge mode IE_B_ in Fig. 4d is the sum distribution of 8 degenerate modes and can be divided into 2 types of modes. One is distributed at the 4 corners, and the other is at the 4 central edges, while the change of each single inner edge mode IE_B_ is still small. When inner edge state IE_B_ is generated by the single-site excitation, the energy of one IE_B_ mainly localizes at its sub square lattice but slowly transports to other sub square lattices corresponding to degenerate modes, which is negligible in a short evolution distance. However, field intensities of the bulk mode with dispersion spread to the whole sites randomly (Fig. 4e). Although there exist deviations of coupling strengths in the fabricated fractal photonic AFTI samples as presented in the table of transmittances of DCs in the Supplementary information, the experimental results in Fig. 3e–g demonstrate that the disorders just induce a slight dispersion and chiral edge states still propagate along the designed trajectory and main energies transport to the designed output lattice sites. That is to say, chiral edge modes in the fractal photonic AFTI are robust against off-diagonal disorders, which are significantly different from diffusible bulk modes.

Furthermore, these chiral edge modes are robust against defects (missing sites). The simulations of the light dynamic evolution (Fig. S8 in the Supplementary information) demonstrate that all chiral edge modes move around a missing lattice site without backscattering or penetrating into the bulk and continue to propagate along newly formed boundaries, confirming the existence of topologically protected chiral edge modes by the single-site excitation^2,3^. Above all, outer edge modes, inner edge modes IE_A_ and IE_B_ in the fractal photonic AFTI are all topologically protected for their unidirectionality and high robustness against various disorders and defects.

Unidirectional topological edge transport can be used to protect fragile quantum states from imperfections in the lattice, enhancing photonic quantum technologies with topological properties. Quantum Hong-Ou-Mandel interference of single photons at a balanced beam splitter is a key phenomenon in quantum physics and lies at the heart of linear photonic quantum computation^33,34^. The fractal photonic AFTI supports robust and high-efficiency topological edge transport of multiple single-photon chiral edge states, and it is flexible to conduct the quantum interference of arbitrary two of them by constructing a DC to directly connect the two output sites.

**Fig. 5:**
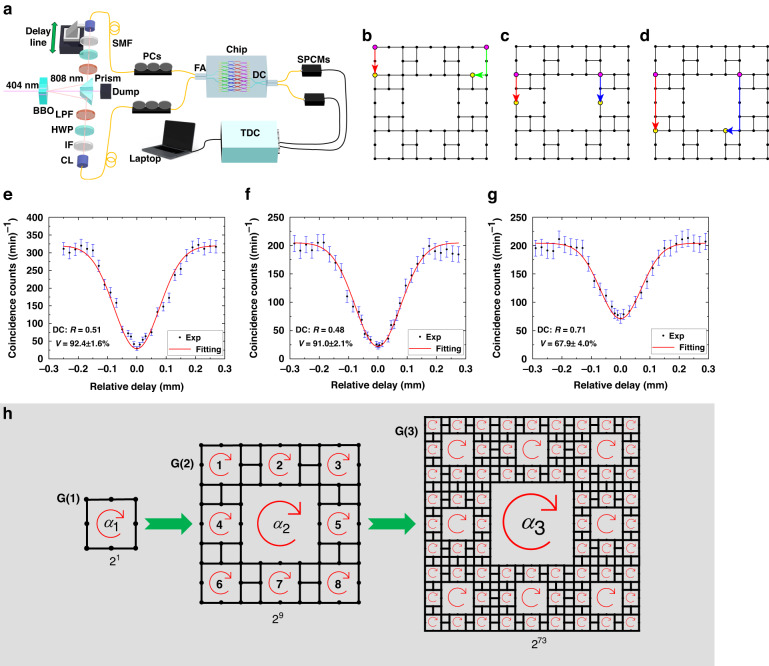
Quantum interference of single-photon chiral edge states in fractal photonic lattice. **a** Experimental setup for characterizing the quantum interference. The DC used for the quantum interference is integrated in the photonic circuit to improve the stability and operability. The quantum source is the Type-I SPDC source. TDC, Time-Digital-Converter. **b**, **c** Quantum outer edge state and inner edge state IE_B_ (IE_A_) are transferred from input sites (pink dots) to output sites (yellow dots) in the one-period lattice. The red, blue, and green arrows represent the unidirectional topological transport of single-photon outer edge, inner edge IE_A_, and inner edge IE_B_ states, respectively. **d** Quantum outer edge state and inner edge state IE_A_ are transferred from input to output sites in the two-period lattice. **e**–**g** The quantum interference visibility *V* of output photons (**b**–**d**) on a DC with a reflectivity of (0.51, 0.48, 0.71) is (92.4 ± 1.6%, 91.0 ± 2.1%, 67.9 ± 4.0%). All error bars are calculated by assuming Poisson statistics. **h** Sketch of fractal patterns for the 1st, 2nd, and 3rd generations as high-capacity quantum information carriers. Numbers represent the total amount of quantum states that can be encoded by inner edge states

We launch 808 nm single-photon pairs via the Type-I spontaneous parametric down conversion (SPDC) into the fractal lattice to generate a pair of single-photon chiral edge states with topologically protected quantum correlation at separate lattice boundaries. The quantum interferences after completing their one/two-period topological edge transport are measured, as shown in Fig. 5a. Between the two edge states for quantum interference, one is the outer edge state and the other is the inner edge state IE_A_ or IE_B_. Figure 5b (Fig. 5c) indicates the outer edge state and the inner edge state IE_B_ (IE_A_) after propagating one period for quantum interference, and the interference curve is shown in Fig. 5e (Fig. 5f), whose measured interference visibility *V* is 92.4 ± 1.6% (91.0 ± 2.1%) on a DC with a reflectivity *R* of 0.51 (0.48), which is higher than 90%. Considering the initial interference visibility of quantum source is *V*_i_ = 96.8%, the relative visibility is *V*_r_ = *V*/*V*_i_ ≈ 95%, implying that quantum interference of chiral edge states in our system is almost ideal. In Fig. 5c, two edge states have identical transfer trajectory in one period, so we further conduct the quantum interference of the outer edge state and the inner edge state IE_A_ after propagating two periods, as demonstrated in Figs. 5d, g.

Due to the imperfection of fabrication, the measured reflectivity of the DC for interference is *R* = 0.71 instead of the designed 0.5, but the measured interference visibility is *V* = 67.9 ± 4.0%, very close to the theoretical maximum value *V*_M_ = 70%. Well matched quantum interferences on different DCs provide the potential for generating entanglement and performing quantum logical operations.

The high visibilities of on-chip quantum interferences suggest the successful generation and control of highly indistinguishable multiple single-photon propagating chiral edge states that unidirectionally transport along various lattice boundaries. In addition, single photons undergo quantum interference on the balanced DC can generate biphoton NOON state ($(|20\rangle +|02\rangle )/\sqrt{2}$) due to bunching effect and further propagate as the topologically protected outer/inner edge entangled state via spatial or chiral encoding ($(|oo\rangle +|ii\rangle )/\sqrt{2}$ or $(|\circlearrowleft \circlearrowleft \rangle +|\circlearrowright \circlearrowright \rangle )/\sqrt{2}$) in the subsequent lattice with proper theoretical design seen in Supplementary Information, where $|o\rangle$($|i\rangle$) represents the outer (inner) edge state and $|\circlearrowleft \rangle$ ($|\circlearrowright \rangle$) represents the anticlockwise (clockwise) edge state. Furthermore, the generated indistinguishable single-photon chiral edge states can be used to encode quantum information to perform quantum logical operations, such as undergoing quantum interference on a DC with *R* = 1/3 to realize the control Z (CZ) operation^13,35^.

When multiple chiral edge states are simultaneously excited by single photons with quantum correlation, the fractal photonic lattice serves as a stable high-capacity quantum information carrier with topological protection via high-dimensional encoding. With higher generations of fractals, the encodable quantum information capacity of a fractal pattern increases exponentially. As shown in Fig. 5h, only considering the existence (1) and non-existence (0) of inner edge modes, numbers of total amount of quantum states that can be carried by G(1), G(2) and G(3) are 2, 2^9^, and 2^73^, respectively. If using the outer edge mode at the same time, the information capacity of each generation doubles. When injected single photons are in superposition or entanglement, the quantum information capacity is further enlarged, and the fractal photonic AFTI can even play as a robust quantum information processing platform with a powerful quantum parallel computing capability.

Thanks to the self-similarity, the fractal AFTI lattice has the advantages of the well-defined symmetry, geometrical scalability, and internal connectivity, which can’t be realized by the AFTI lattice with randomly missed bulk sites though it can also preserve outer edge modes and support inner edge modes associated with the inner boundaries of lattice holes because the path taken by wave packets in the AFTI is explicitly defined by the discrete driving protocol. Firstly, the fractal AFTI lattice has a well-defined symmetry that includes the specific holes at different generations due to self-similarity, but the random lattice spoils the symmetry^19^. Secondly, the fractal AFTI lattice exhibits excellent geometrical scalability in producing higher generations through its iteration procedure, which can provide a scalable and controllable mode encoding method to meet the requirement of the scalable multistate topological quantum system and the large-scale linear optical quantum computation. Based on the self-similarity, the fractal dimension of the lattice with dual Sierpinski carpet structure can maintain 1.89D at every generation and the fractal dimension of total chiral edge states is also 1.89D when the number of generations tends to infinity^20,21^, but the random lattice without well-defined removal rules can’t guarantee this feature. Thirdly, the fractal AFTI lattice retains a systematically connected internal structure by virtue of the hierarchy of square holes^18^, but the fragmented structures of random lattices destroy the internal connectivity, and even produce some isolated sites, resulting in the waste of lattice sites. As shown in the Supplementary information, in a lattice with 17 randomly missed bulk sites, the robustness of some edge modes against the small deviation of coupling strength is weaker than that of the fractal AFTI lattice.

## Discussion

To conclude, we have experimentally realized the fractal photonic AFTI based on DSC structure using FLDW and performed on-chip quantum interferences of multiple propagating single-photon chiral edge states existing in the fractal lattice with high interference visibilities for the first time. By introducing fractals into AFTIs, both the type and the number of chiral edge modes in a single photonic lattice increase significantly, providing an approach to efficient manipulation of multistate topological quantum systems. These topological chiral edge modes protect the quantum correlations of injected single-photon pairs during the quantum state transport in the fractal photonic AFTI lattice and make the measurement of HOM interference easy to conduct. The high interference visibilities verify that single-photon chiral edge states generated at various lattice boundaries are highly indistinguishable, which are required in encoding quantum information to perform quantum logical operations or generate topologically protected quantum entanglement resource. With the growth of fractal generations, the emerging multiple inner edge modes integrated with quantum source and high-dimensional encoding enlarge the encodable quantum information capacity carried by a single photonic lattice substantially. These fractal and topological advantages provide the potential to enhance multi-fold manipulation of quantum states in a single lattice, such as supposition, entanglement, and quantum information processing. This work lays the foundation for the scalable topological photonic quantum computation based on multiphoton states, quantum simulation of multiparticle systems and high-capacity quantum information transmission via high-dimensional encoding. What’s more, this fractal lattice as a platform for efficient quantum simulation can be applied to exploration of quantum effects in real topological condensed materials that are elusive to observe, and to finding more novel physical phenomena to provide guidance on the theory and experiment. In addition to the Sierpinski carpet and Sierpinski gasket, the underlying physical laws can extend to many other fractal structures, which can broaden the field of fractal photonic TIs.

## Materials and methods

### Sample fabrication

The waveguides constructing the fractal photonic lattice inside the borosilicate glass (Eagle2000, Corning) were fabricated by the femtosecond laser direct writing. The 1030 nm pulses from a regeneratively amplified Yb: KGW laser (Pharos-20 W-1 MHz, Light Conversion) with the duration of 240 fs and the repetition rate of 1 MHz was focused below the surface of the glass by a microscope objective (NA 0.5, 20×). The depth ranges from −30 μm to −270 μm with a spacing of 30 μm. The motion of the sample was controlled by an air-bearing three-axis high-precision positioning stage (FG1000, Aerotech,). The total length of the glass sample is 70 mm. By optimally selecting the pulse energy of 470 nJ and the scanning speed of 20 mm/s, all the fabricated waveguides work in single mode with similar performances. The extra power compensation by the acoustic optical modulator and the correction of spherical aberration by the spatial light modulator are not applied, because it is hard to synchronize due to the fast scanning speed. Although the cross sections of waveguides fabricated in different depths indeed have slight variations, as shown in Fig. S16 in the Supplementary information, they don’t dramatically influence experimental results.

### Lattice parameters

The fractal photonic lattice is a 9 × 9 lattices with 64 sites. The site spacing is set as 30 μm to avoid undesired coupling between adjacent waveguides and reduce the length of lattice as much as possible. For each step in one period, there are 22 directional couplers with a transmissivity of about 100%, whose interaction distances are 8 μm and 10 μm for horizontal and vertical coupling, respectively. The length along the direction of propagation for the S bending is 2 mm. For the horizontal coupling, the interaction lengths are 1.10, 1.30, 1.20, 1.20, 1.10, 1.40, 1.50, 1.70, 1.30 mm from the top to the bottom layers, respectively. For the vertical coupling, the interaction lengths are 1.80, 2.20, 2.20, 2.20, 2.60, 1.90, 2.40, 2.00 mm from the top to the bottom gaps, respectively. Transmissivity for each DC is included in Supplementary Information. As for the DCs for quantum interference, the interaction distance is 8 μm, the interaction length is 0.55 mm and the pitch of output ports is set as 127 μm to match the fiber array.

### Quantum characterization

The 808 nm two-photon source is generated by pumping a 0.5-mm-thick Type-I beta-barium borate (BBO) crystal using 404 nm CW laser. Using long pass filters (>650 nm) and 3-nm interference filters (IF, 808 nm) to guarantee the indistinguishability of spectrums. Half wave plates (HWPs) and polarization controllers (PCs) are used to ensure input photons are in vertical polarization and guarantee the indistinguishability of polarization modes. The delay line is used to adjust the arrival time of photons at DCs for quantum interference to maintain the temporal indistinguishability. The initial quantum interference visibility of the source on a balanced fiber beam splitter (FBS, 1:1) is *V*_i_ = *V*_FBS_ = 96.8 ± 0.3%, as shown in supplementary information. For measuring convenience, we inject photons into the lattice sites at the same layer and collect photons output from lattice sites at the same layer, too. To match the pitch of input fiber array, two S bends are used to connect the input ports with a spacing of 127 μm and the lattice sites for injection. The DCs for quantum interference are integrated in the chip, and the spacing of their output ports is 127 μm to match the output fiber array. The collected output photons are sent to the single-photon count modules (SPCMs) by single mode fibers (SMFs) and the detection signals are conveyed to the time-to-digital converter (TDC) to conduct coincidence measurements.

### Supplementary information

Supplementary Information for Fractal photonic anomalous Floquet topological insulators to generate multiple quantum chiral edge states
