# Supplementary material for: Fractal photonic anomalous Floquet topological insulators to generate multiple quantum chiral edge states
Source: Light Sci Appl. 2023 Nov 2;12:262. doi: 10.1038/s41377-023-01307-y (PMC10620381; doi:10.1038/s41377-023-01307-y)
Supplement: Supplementary file 1 — Supplementary Information for Fractal photonic anomalous Floquet topological insulators to generate multiple quantum chiral edge states [file 41377_2023_1307_MOESM1_ESM.docx]

**Supplementary Information for**

**Fractal photonic anomalous Floquet topological insulators to generate multiple quantum chiral edge states**

Meng Li^1, 2, *^, Chu Li^1, 2^, Linyu Yan^1, 2^, Qiang Li^1, 2^, Qihuang Gong^1, 2, 3, 4, 5^ and Yan Li^1, 2, 3, 4, 5, *^

^1^State Key Laboratory for Artificial Microstructure and Mesoscopic Physics, School of Physics, Peking University, Beijing 100871, China.

^2^Frontiers Science Center for Nano-Optoelectronics, Peking University, Beijing 100871, China.

^3^Collaborative Innovation Center of Extreme Optics, Shanxi University, Taiyuan, Shanxi 030006, China.

^4^Hefei National Laboratory, Hefei 230088, China

^5^Peking University Yangtze Delta Institute of Optoelectronics, Nantong 226010, China.

* Corresponding author: [mengli2016@pku.edu.cn](mailto:mengli2016@pku.edu.cn); [li@pku.edu.cn](mailto:li@pku.edu.cn).

1. **Sierpinski carpet lattice**

**1.1 Degenerate modes**

The quasienergy spectrum of the fractal photonic anomalous Floquet topological insulator (AFTI) using the dual Sierpinski carpet (DSC) lattice demonstrates several degenerate modes, which are inner edge modes IE_B_ and bulk modes. Each quasienergy of the inner edge mode IE_B_ has 8 degenerate modes, whose field intensities are demonstrated in Fig. S1. The 8 degenerate modes locate at each G(1) lattice (sub square) and exhibit similarity in distribution. Four of them are at the corners (IE_B_-Type I, Fig. S1a, c, e, g) and the other four are at the central edges (IE_B_-TypeII, Fig. S1b, d, f, h). Bulk modes are corresponding to 16 degenerate zero modes. The field intensities of 16 dispersionless bulk modes are shown in Fig. S2, which are the same with their excitation sites. After a single-period evolution, the light comes back to its initial site. The evolution operator over one period of the drive is trivial ($\hat{U}(z_{0})=1$) in the bulk. The sum distributions of inner edge modes IE_B_ and bulk modes are shown in Fig. S3a and b, respectively. As can be seen, the introduction of fractal structure reduces the number of bulk modes and produces more inner edge modes for the photonic AFTI.


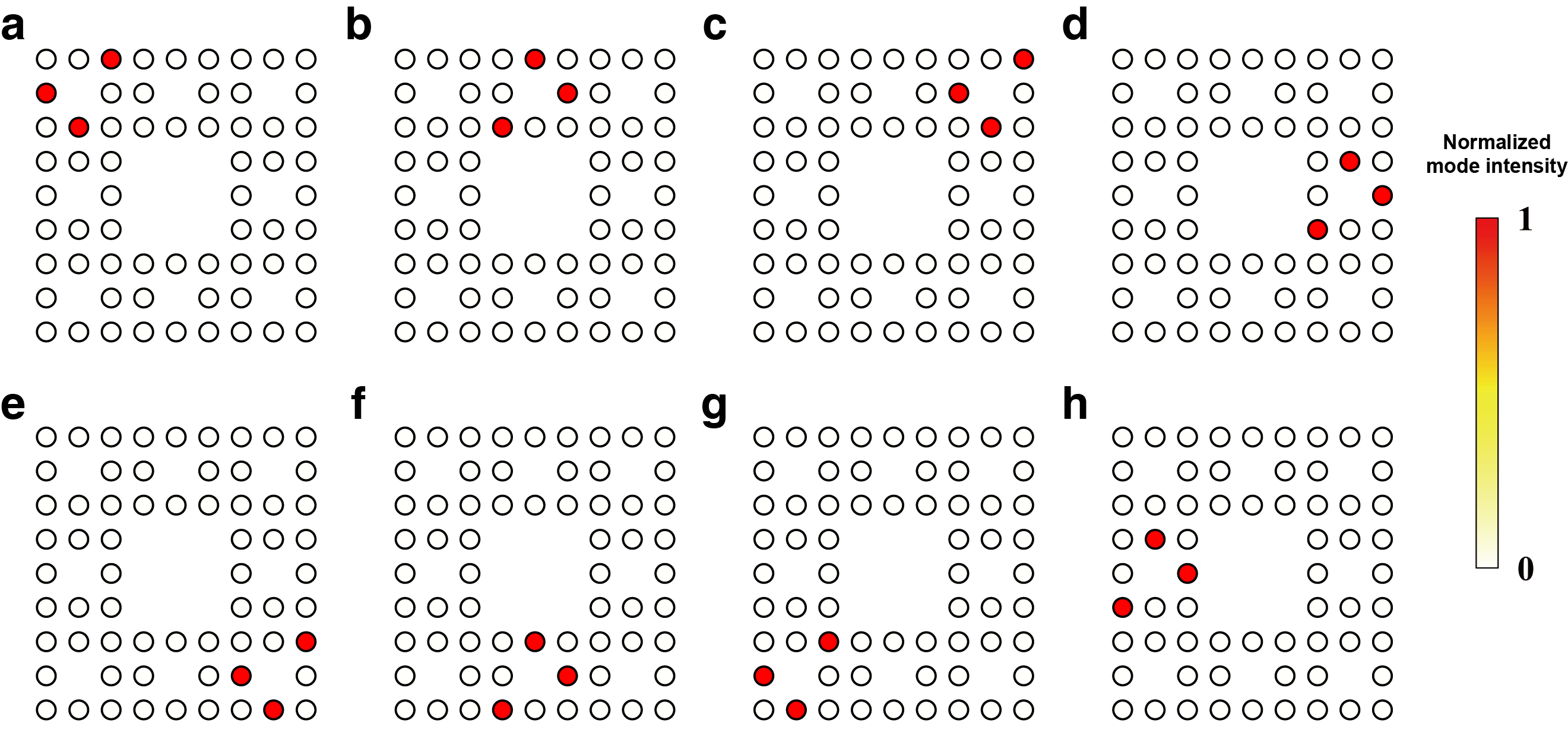


**Fig. S1 | Eight degenerate inner edge modes IE_B_ of the fractal AFTI in the Sierpinski carpet lattice: a-h.**


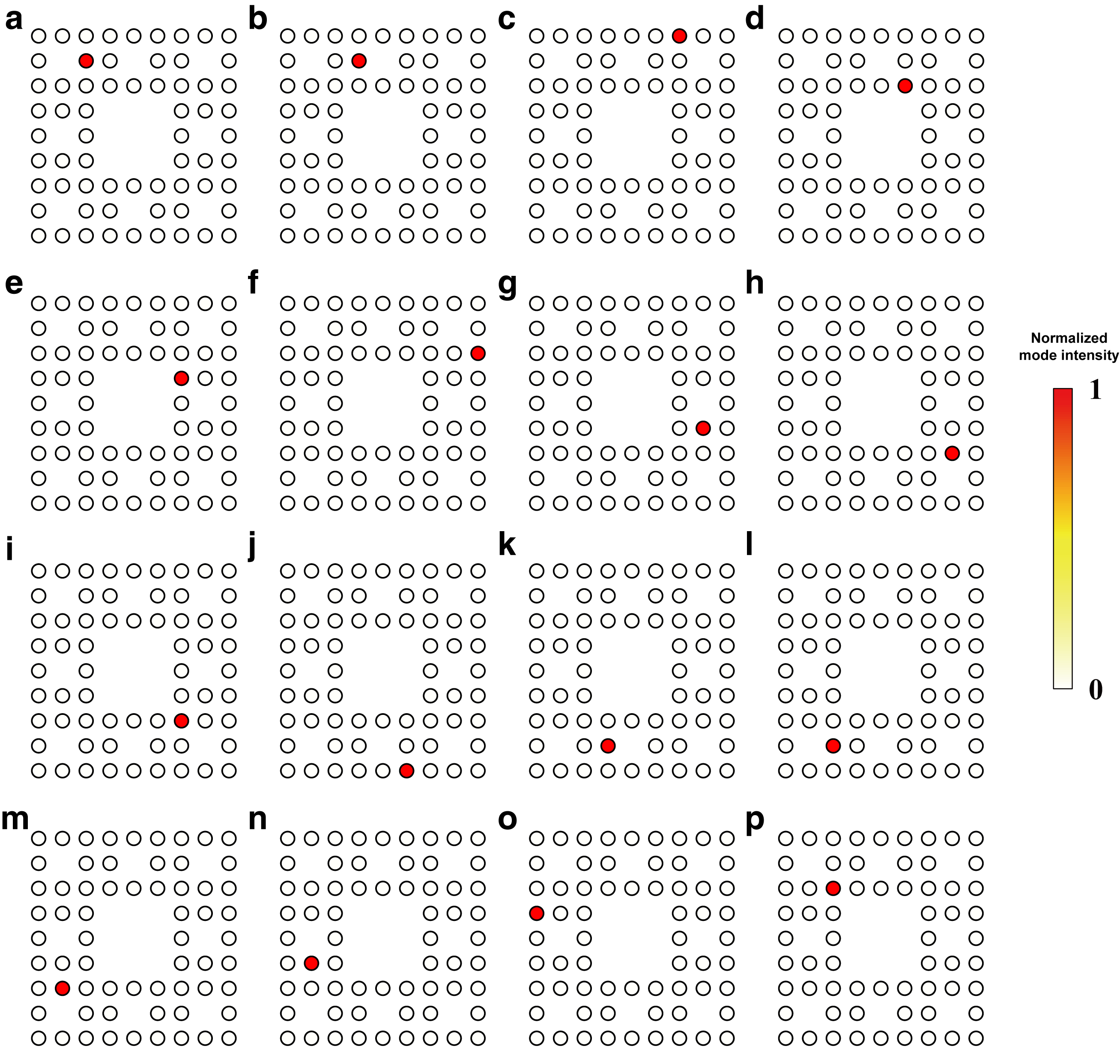


**Fig. S2 | Sixteen** **dispersionless bulk modes of the fractal AFTI in the Sierpinski carpet lattice: a-q.**

**
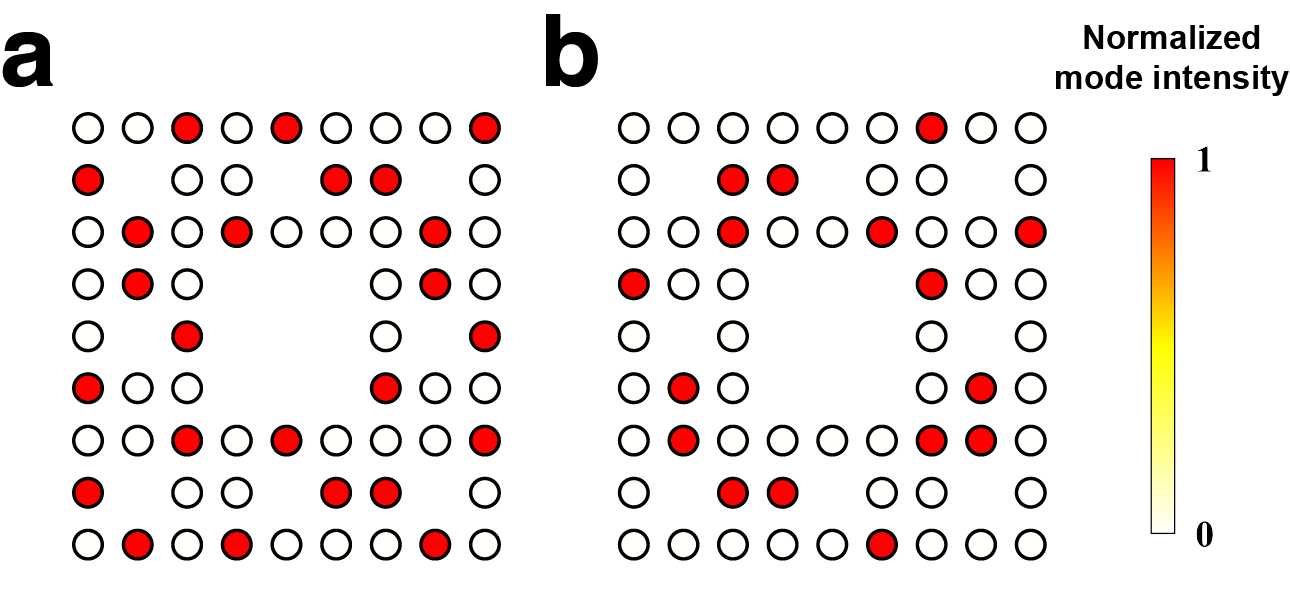
**

**Fig. S3** | Sum distribution of field intensity in the fractal lattice. **a,** Inner edge modes IE_B_. **b,** Bulk modes.

**1.2 Robustness against the deviation of coupling strength**

It should be noted that there still exist outer edge modes, inner edge modes IE_A_ and IE_B_ when the quasienergy is zero. Considering the deviation of coupling strength $\Lambda_{0}$ in a proper range, we find that quasienergies of the outer edge mode and the inner edge mode IE_A_ can still maintain near zero, but those of some inner edge modes IE_B_ change a lot and these modes become dispersive bulk modes. When the deviation of coupling strength $\delta(\Lambda)/\Lambda_{0}$=0.15, the quasienergy spectrum of the new fractal photonic lattice is shown in Fig. S4. The red dots, blue dots, green dots, and black dots represent outer edge modes, inner edge modes IE_A_, inner edge modes IE_B_ and bulk modes, respectively.

For the inner edge modes IE_B_ represented by green dots, the original flat degenerate quasienergy spectrum becomes a little dispersive, and the field intensities demonstrate a sum distribution of inner edge modes IE_B_, as shown in Fig. S5. The mode of No.9 can be seen as the sum of that of No. 8 and No.10. Though with deviation, there still exist four zero points (No.31-No.34) at the mid of the quasienergy spectrum as shown in Fig. S4. The field intensities of these four points are shown in Fig. S6. No. 31 stands for the inner edge mode IE_A_ and No. 33 is the outer edge mode, while No.32 and No.34 represent two dispersive bulk modes. As for the inner edge modes IE_B_ with zero quasienergy in the lattice without deviation, they are transformed into dispersive bulk modes in this lattice with deviation. From the slight changes of quasienergy spectrum and the distribution features of field intensities, the edge modes represented by the red, blue, and green dots are all topologically protected.


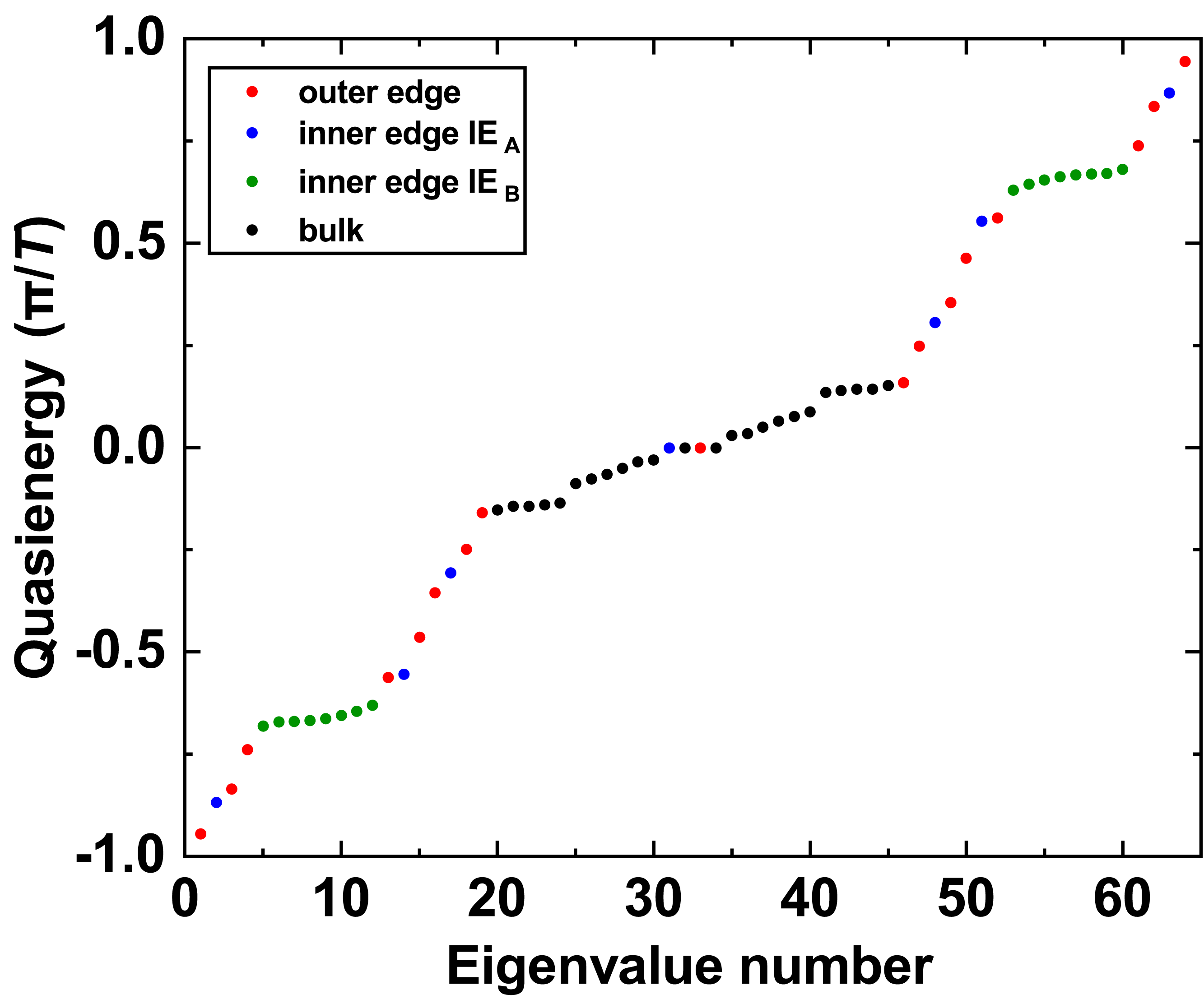


**Fig. S4 |** **Quasienergy spectrum for the 64-site fractal photonic lattice** **when the** **deviation of coupling strength is 0.15.** The black dots represent the bulk modes, and the red, blue, and green dots represent outer edge, inner edge modes IE_A_ and IE_B_, respectively.


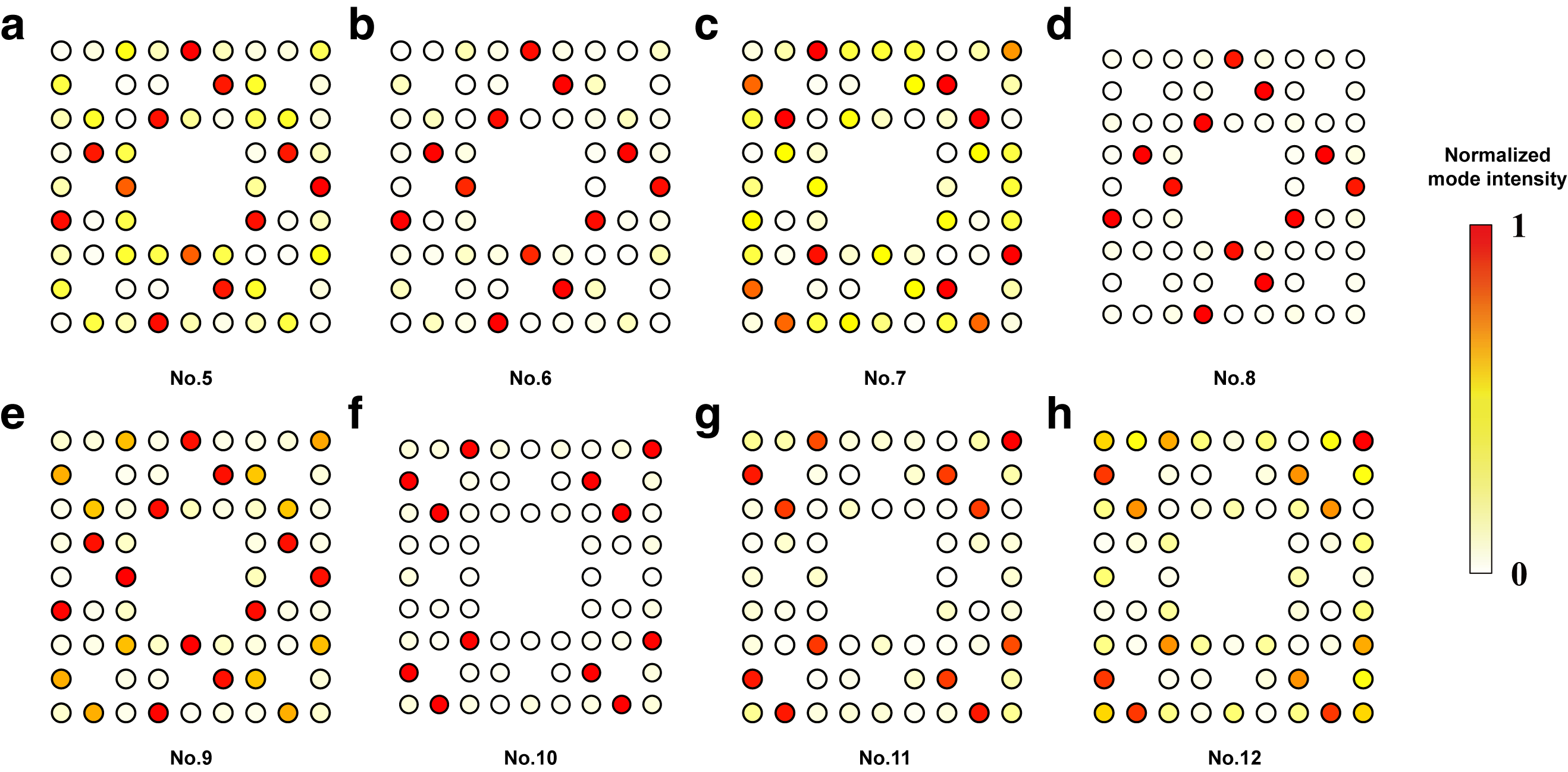


**Fig. S5 |** **The field intensities of the inner edge modes IE_B_ are shown for eigenvalue number ranging from No.5-No.12,** **when the deviation of coupling strength is 0.15.**


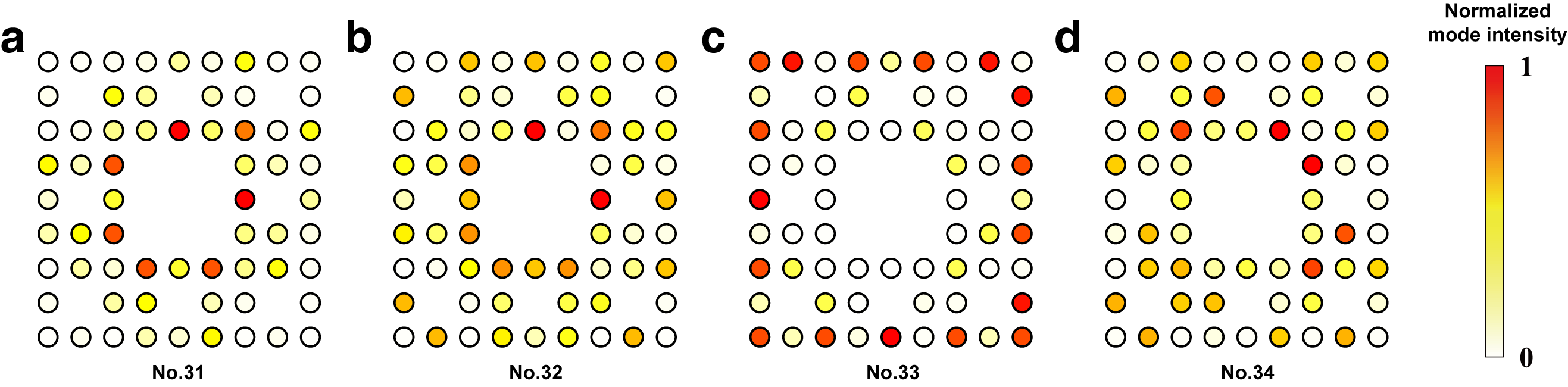


**Fig. S6 | The field intensities of the four modes with zero quasienergy when the deviation of coupling strength is 0.15.** The eigenvalue numbers are No.31-No.34. No.31 and No.33 represent the inner edge mode IE_A_ and the outer edge mode, respectively. While No.32 and No.34 are both dispersive bulk modes.

By simulation of the dynamical evolution of various edge states generated by the single-site excitation in the normal and fractal AFTI lattices with the deviation of coupling strength, their surrounding lattice sites mainly served as the “bulk” region are different.

The outer edge states in the fractal AFTI maintain the same field intensity distributions and transport behavior as the usual edge states in the normal AFTI, but its robustness against disorders is a little weaker due to the replacement of some surrounding bulk sites by lattice sites corresponding to inner edge states IE_B_. The inner edge states IE_B_ can trap more energy of the light escaped from the outer edge state for a long evolution distance than the bulk state, resulting in a relatively larger amplitude oscillation of the total field intensity distribution (*P*) of the outer edge state than that of the usual edge state, as shown in Fig. S7.

For outer edge states and inner edge states IE_A_ in the fractal AFTI, they have the similar surrounding lattice sites mainly served as their “bulk” region, located inside the outer edges but outside the inner edges of the central hole. For inner edge states IE_B_-Type I and IE_B_-Type II, the surrounding lattice sites possessed by outer edge states and bulk states mainly serve as the “bulk” region, but the surrounding lattice sites possessed by inner edge states IE_A_ can also serve as the “bulk” region for IE_B_-Type II.


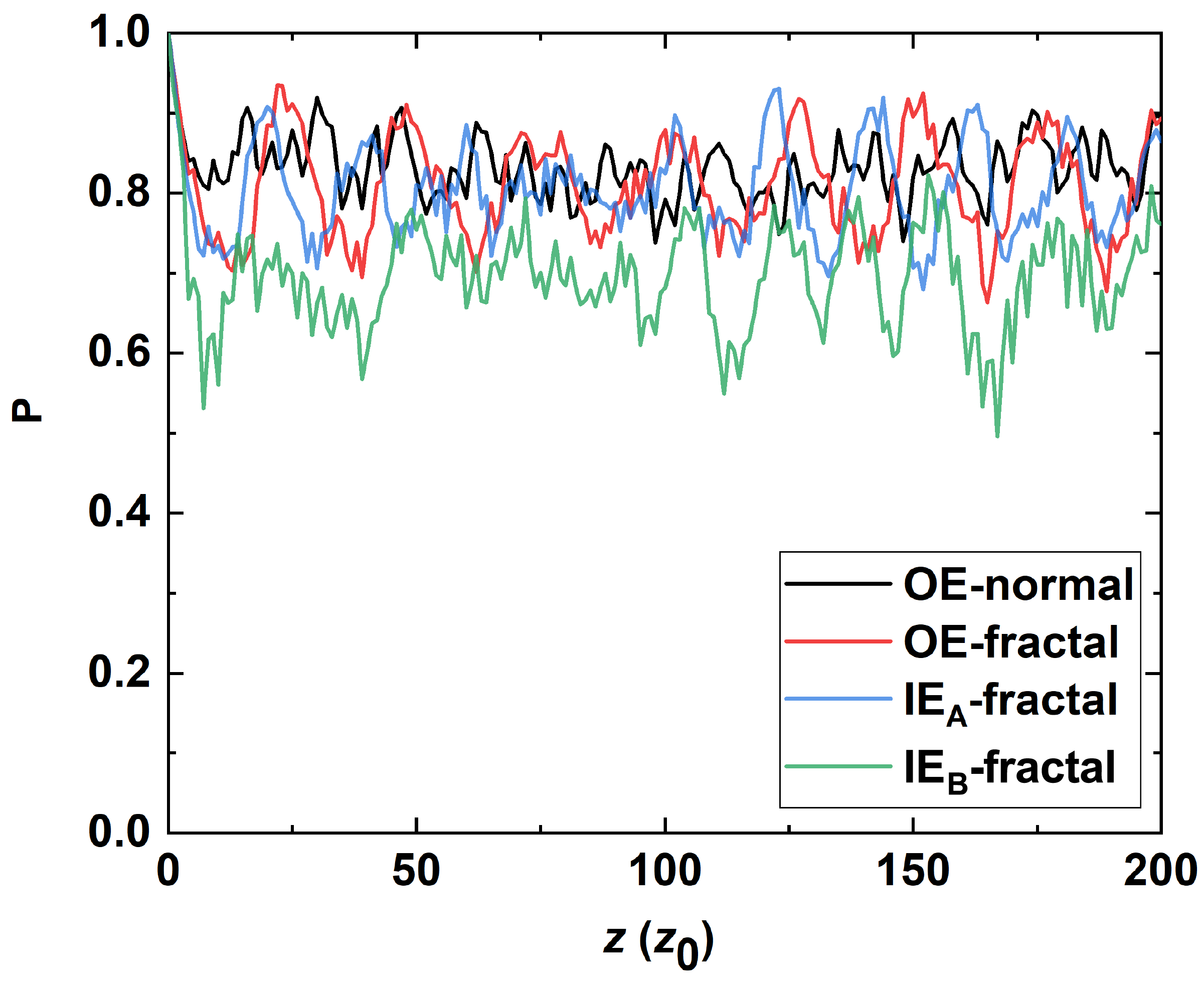


**Fig. S7 |** **The variation of total field intensity distributions (P) of various edge states with the evolution distance z when the deviation of coupling strength *δ*(Λ)/Λ_0_=0.1, which reflects the strength of robustness against disorders.** The robustness of the outer edge state is close to that of the inner edge state IE_A_, and stronger than that of the inner edge state IE_B_, but a little weaker than that of the usual edge state in the normal AFTI lattice.

When there exist disorders (deviations) of coupling strength, main energies of outer edge states and inner edge states IE_A_ can remain at their corresponding edges of the lattice with minimal penetrating into the “bulk” region, and the two kinds of edge states show similar robustness against disorders. However, due to the existence of degenerate modes, the energy of one inner edge state IE_B_ mainly localizes at its sub square lattice but slowly transports to other sub square lattices, so the robustness of inner edge state IE_B_ is weaker, as shown in Fig. S7.

**1.3 Robustness against defects**

We demonstrate the light dynamical evolution of three kinds of chiral edge modes in the fractal photonic lattice with defects (marked by ×), as shown in Fig. S8. Figure S8a and S8b demonstrate the propagation of chiral outer edge mode in the fractal photonic lattice with missing sites at the outer periphery. Figure S8c demonstrates the propagation of chiral inner edge mode IE_A_ in the fractal photonic lattice with one missing site at the corner of the inner periphery. Figure S8d demonstrates the propagation of chiral inner edge mode IE_B_ in the fractal photonic lattice with one missing site in the bulk. Although introducing defects breaks the self-similarity of the fractal AFTI lattice, all simulation results confirm that these chiral edge modes can move around the missing lattice site without scattering or penetrating in the bulk and continues to travel along newly formed boundaries.


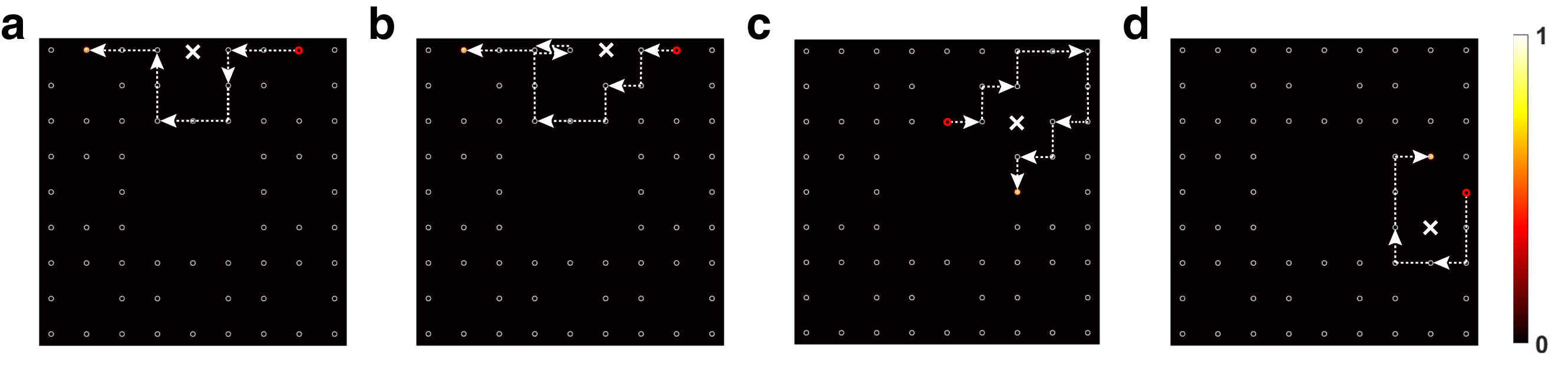


**Fig. S8 | The simulation of the propagation of various chiral edge modes in the lattice, which are robust against defects (missing sites marked by ×).** The red circles mark the injection sites. The white dash arrows describe the energy flow in the lattice, and each arrow is corresponding to an evolutional distance of z_0_. **a** and **b**, The outer edge mode transfer in the lattice with a defect at the outer periphery. **c,** The inner edge mode IE_A_ transfer in the lattice with a defect at the corner of the inner periphery. **d,** The inner edge mode IE_B_ transfer in the lattice with a bulk defect.

**1.4 Difficulty in determining topological invariant**

The fractal photonic AFTI lattice is an aperiodic system, so it is not applicable to use the conventional energy band theory for the periodic system to calculate the topological invariant, such as Chern number and winding number^1^. We get the band structure of a fractal lattice according to the conventional energy band theory. To form a periodic strip geometry along x direction, two G(2) lattices are linked together according to the driving protocol as a unit cell with 128 lattice sites, which are shown in Fig. S9a. The quasienergy spectrum of the unit cell lattice is shown in Fig. S9 b, which is like that of one G(2) lattice. The number of inner edge modes IE_A_ and IE_B_ is doubled, which is the sum of two sub lattices. The number of outer edge modes also increases, and modes are distributed at the newly formed larger outer boundary of the lattice. The Floquet spectrum (quasienergy band) of the lattice with standard coupling strength Λ_0_=π/2 is shown in Fig. S9c, which is much more complex than that of a normal lattice^2, 3^. However, the displayed band structure can just demonstrate multiple topological outer edge modes (oblique lines), and the group velocity (slope) of outer edge modes is the same with that of a normal lattice. However, bands of all inner edge modes are regarded as bulk bands (flat bands) in this method, which can’t describe the topological properties of inner edge modes due to the lack of extended boundaries. When there exists the deviation *δ*(Λ)/Λ_0_=0.15, many bands of inner edge modes are dispersive, as shown in Fig. S9d. Therefore, this conventional method is unable to determine the topological invariant (winding number) of newly generated inner edge modes in fractal AFTIs correctly.


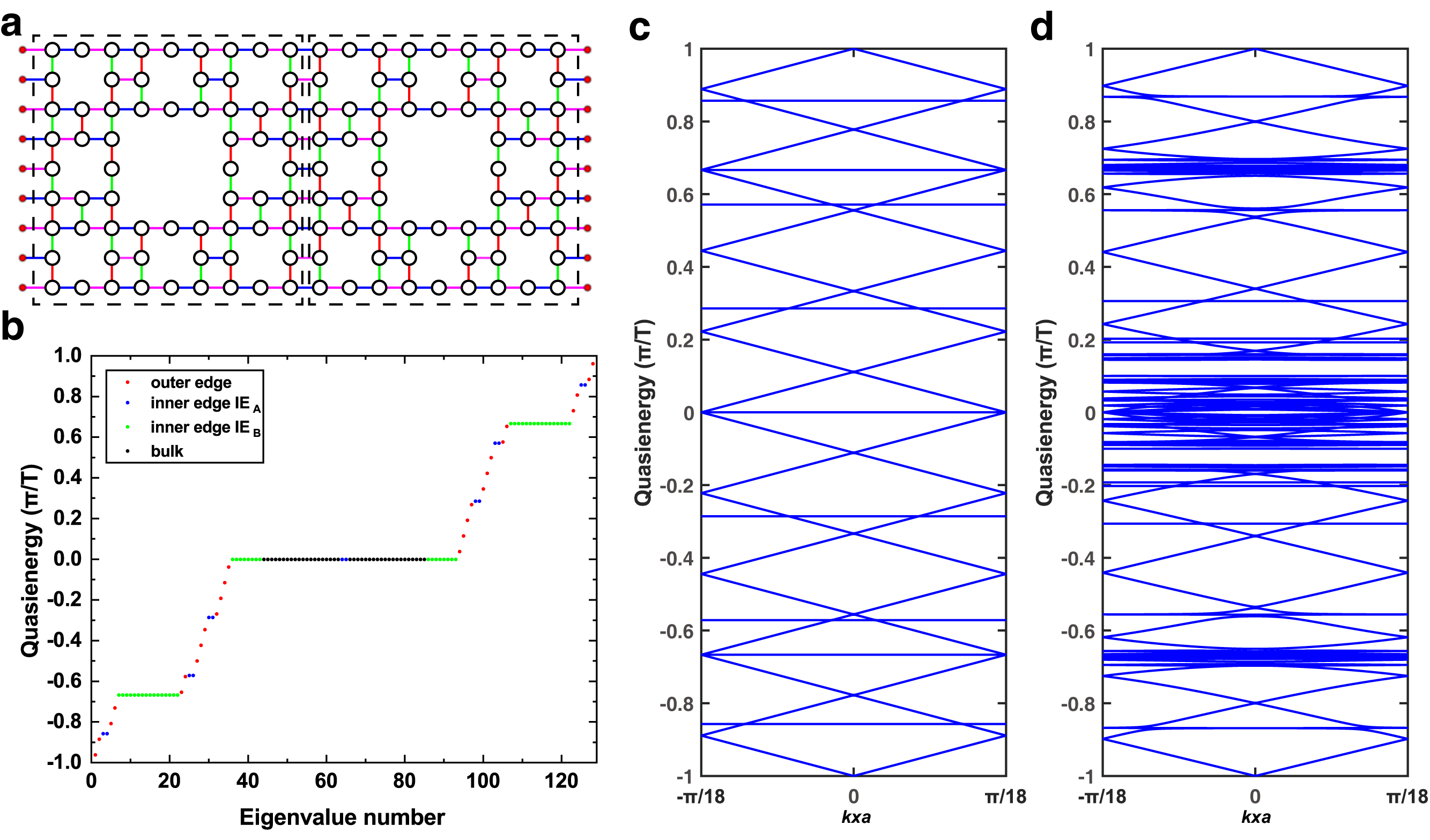


**Figure. S9| Quasienergy spectrums and band structures of the fractal photonic AFTI. a,** Two G (2) lattices are linked together as a unit cell to form a periodic strip geometry along x direction. **b,** Quasienergy spectrum of the unit cell with 128 sites. **c,** Floquet spectrum calculated considering the strip geometry in **a** when Λ=π/2. **d,** Floquet spectrum calculated considering the strip geometry in **a** when$\delta(\Lambda)/\Lambda_{0}=0.15$.

In addition, to verify the edge mode in their fractal lattice are indeed topological, previous works employ the real-space-Chern number as topological invariant for the fractal Floquet topological insulator constructed by helical waveguides^4-6^. Considering that the Chern number of the AFTI is zero^1-3^, the real-space Chern number is still zero. Besides real-space Chern number, the Bott index is also used to describe the topological invariant for the nonperiodic system, such as topological photonic quasicrystals^7^. However, it is still invalid for the fractal AFTI. In analogy with real-space Chern number, there are some works about real-space winding number for disordered system, but all of them are limited to one dimension^8-11^. Maybe in the future, a better method to determine the topological invariant for the fractal AFTI may be found.

**1.5 Quasienergy spectrum of G(3)**

With the increase of the generation, the number of chiral edge modes carried by a single fractal lattice increases significantly. The fractal photonic AFTI based on DSC structure at G(3) is a huge lattice composed of 512 sites (Fig. S10a) and individual couplings are controlled by the 4-step perfect hopping driving protocol. According to the quasienergy spectrum displayed in Fig. S10b, the G(3) lattice support 53 outer edge modes, 56 inner edge modes IE_A_, 192 inner edge modes IE_B_, 19 inner edge modes IE_C_ and 192 bulk modes, and field intensities of these modes are shown in Fig. S10c-g, respectively. Similarly, inner edge modes IE_A_ and IE_B_ also have many degenerate modes. Inner edge modes IE_C_ are newly generated chiral edge modes. The total number of chiral edge modes in a G(3) lattice is 320. The three inner edge modes are distributed at various inner boundaries of the lattice without mutual disturbance. It is obvious that inner edge modes IE_A_ and IE_C_ are topologically protected because of rich surrounding bulk sites. Inner edge modes IE_B_ have fewer surrounding bulk sites, but they are also topologically protected.

When there exist a deviation of coupling strength (0.15) in the G(3) lattice (Fig. S11a), apart from outer edge modes (Fig. S11b), inner edge modes IE_A_ (Fig. S11d) and IE_C_ (Fig. S11c), part of inner edge modes IE_B_ also demonstrate nontrivial distributions of field intensities (Figs. S11e-k), which are quite different from disordered bulk modes whose most energies diffuse into the lattice (Figs. S11l-p). Besides distributions of modes shown in Figs. S11e-k, there are also many other inner edge modes IE_B_ with sum distributions of them by further analyzing the quasienergy spectrum.


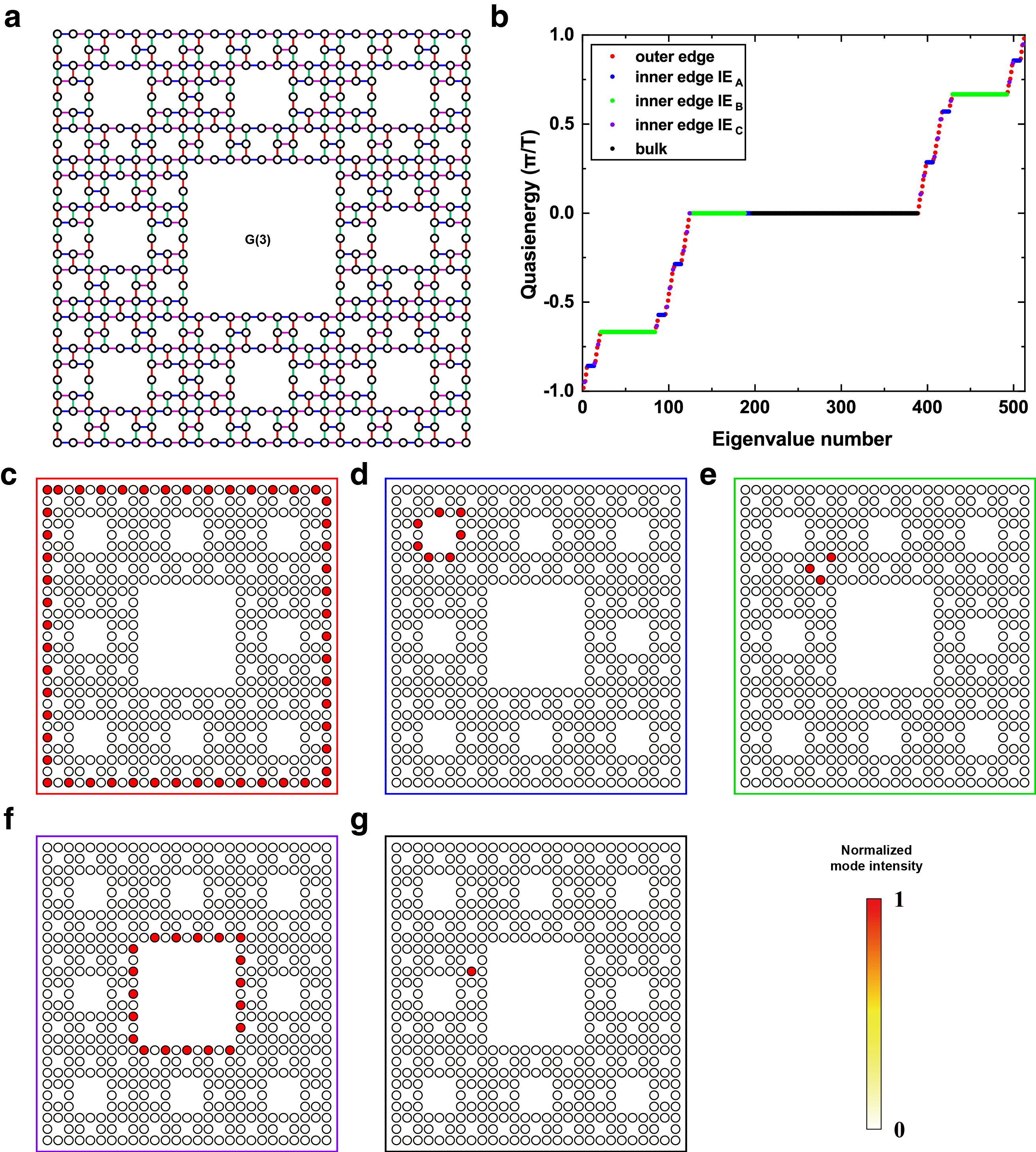


**Fig. S10 | Quasienergy spectrum of fractal photonic AFTI based on DSC structure at G(3).** **a,** Fractal photonic lattice with 512 sites based on DSC structure at G(3). **b,** Quasienergy spectrum of the fractal photonic AFTI at G(3), including 5 modes: outer edge modes, inner edge modes IE_A_, IE_B_, IE_C_, and bulk modes. **c-g,** The field intensities of outer edge modes, one of inner edge modes IE_A_, one of inner edge modes IE_B_, inner edge mode IE_C_, and one of bulk modes.


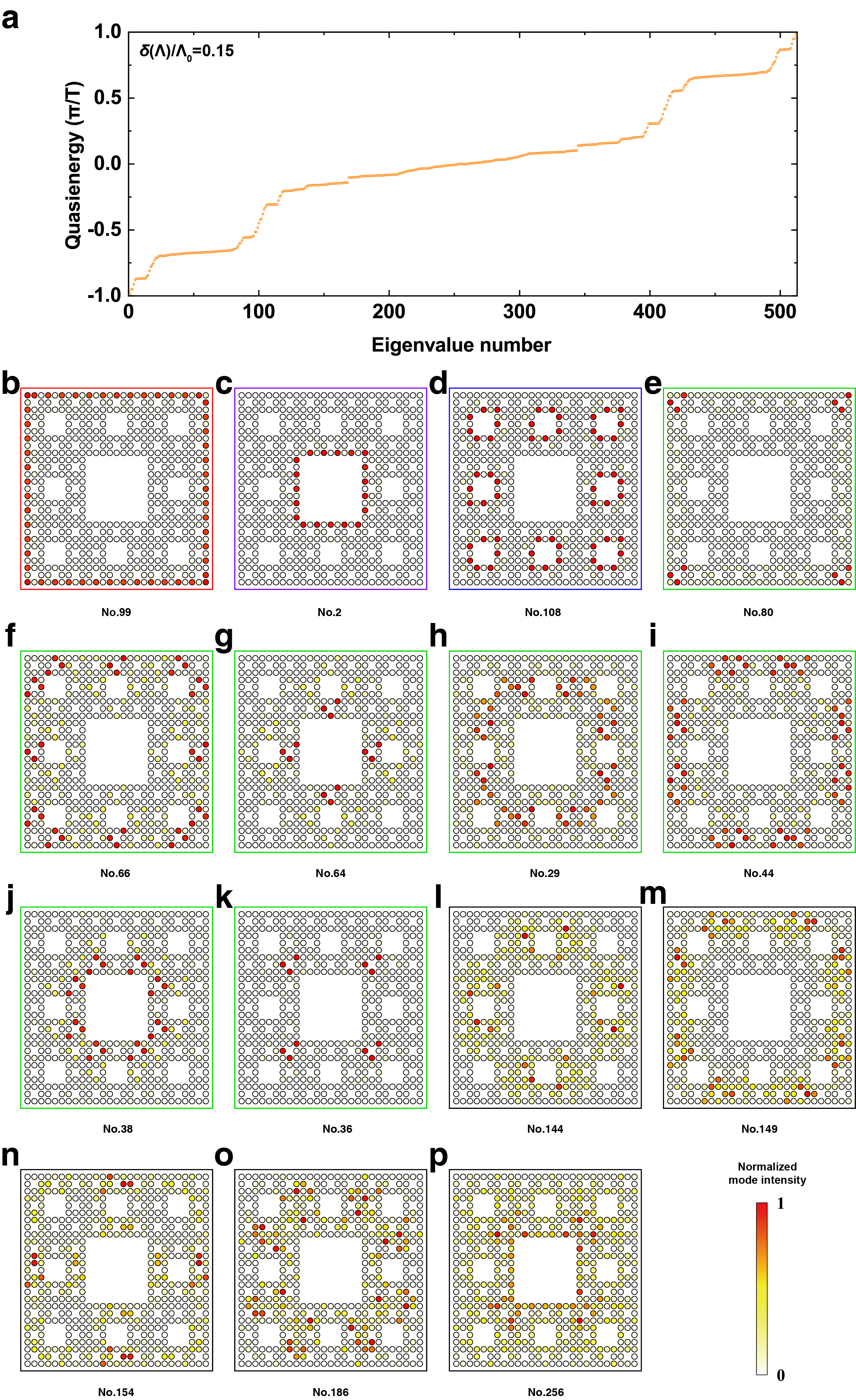


**Fig. S11 | Quasienergy spectrum and field intensities of modes for the 512-site fractal photonic lattice at G(3) when the deviation of coupling strength is 0.15. a,** Quasienergy spectrum of the G(3) lattice with a deviation of coupling strength (0.15). **b, c** Field intensities of outer edge modes and inner edge modes IE_C_. **d,** Field intensities of one of inner edge modes IE_A_. **e-k,** Field intensities of partial inner edge modes IE_B_. **l-p,** Field intensities of partial bulk modes.

1. **Sierpinski gasket lattice**

In addition to the Sierpinski carpet, the Sierpinski gasket can also be combined with the AFTI using the three-step model. The inset of Fig. S12a demonstrates the fractal tringle structures of G(1) and G(2), with a Hausdorff dimension of *d*_f_=ln(3)/ln(2)≈1.585. The G(2) is a 24-site photonic lattice, and its quasi-energy spectrum is shown in the main body of Fig. S12a. There are 13 outer edge modes, 5 inner edge modes IE_A_ and 6 inner edge modes IE_B_. It is interesting that there is no bulk mode, which goes beyond the confine of the bulk-boundary correspondence.


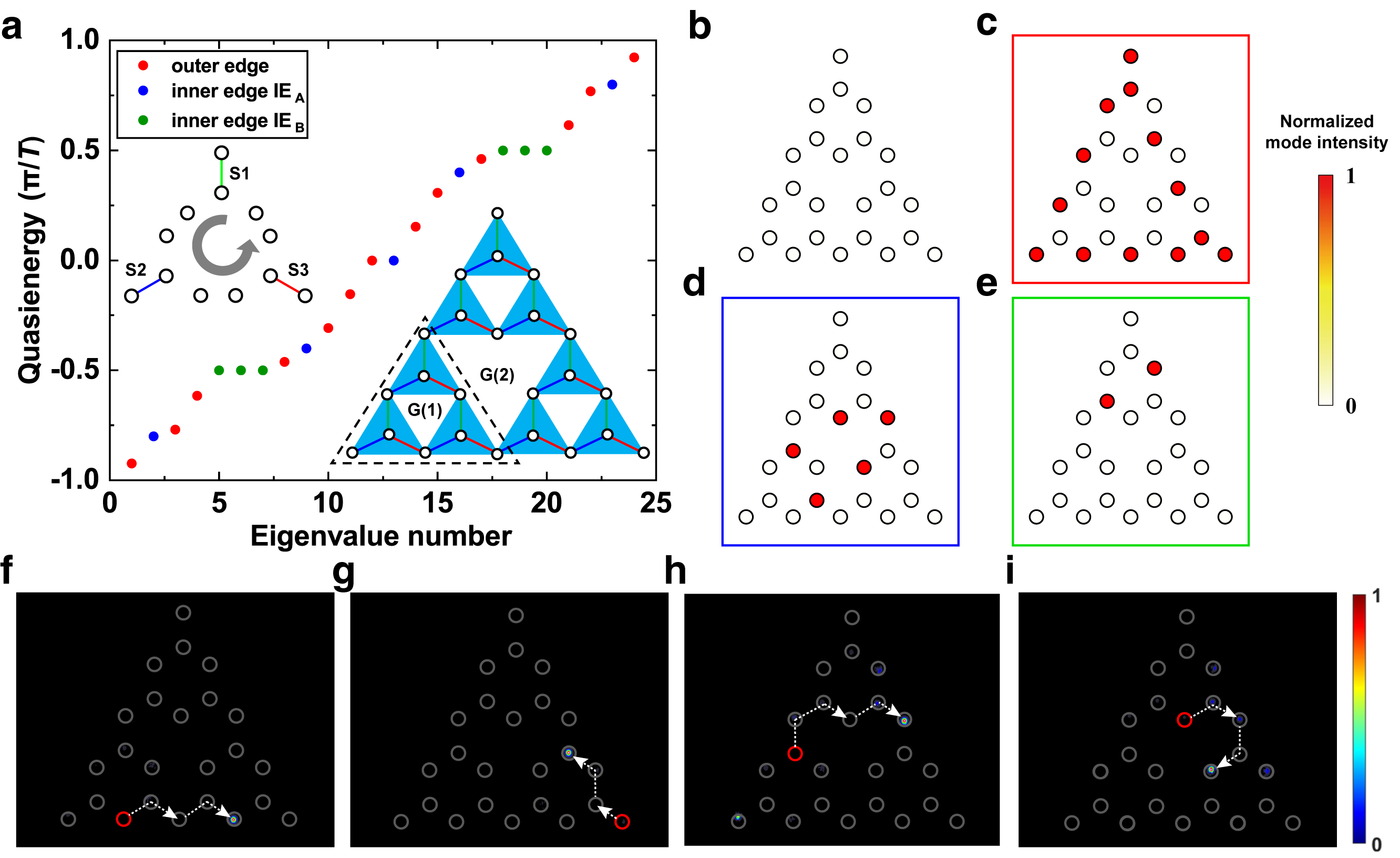


**Fig. S12 | Fractal photonic anomalous Floquet topological insulators in Sierpinski gasket lattice.** **a,** Quasienergy spectrum for the 24-site fractal photonic lattice, where red, blue, and green dots represent outer edge modes, inner edge modes IE_A_ and inner edge modes IE_B_, respectively. The inset in the bottom right is the schematic sketch of the G(1) and G(2) in Sierpinski gasket lattice with three-step model (inset at the top left). **b,** Positions of the Sierpinski gasket lattice sites are marked by circles. **c,** The field intensities of the outer edge modes. **d,** The field intensities of the inner edge modes IE_A_. **e,** The field intensities of the inner edge modes IE_B_. Experimentally observed output intensity distribution when the light is injected into the lattice site indicated by the red circle: **f-g,** outer edge modes; **h-i,** inner edge modes IE_A_. The fabricated lattice is in two periods.

Taking the Fig.S12b as reference, the field intensities of outer edge modes, inner edge modes IE_A_, and inner edge modes IE_B_ are shown in Figs. S12c, d and e, respectively. The samples of the fractal AFTI of Sierpinski gasket are also fabricated by the FLDW technique, and the experimental results also demonstrate the light dynamical evolutionary behavior of outer edge modes (anticlockwise) and inner edge modes IE_A_ (clockwise) in two periods, as shown in Figs. S12f, g and Figs. S12h, i, respectively. As for the fractal photonic AFTI in the Sierpinski gasket lattice, its quasienergy spectrum also demonstrates several degenerate modes. The green dots represent the inner edge modes IE_B_, which have three degenerate modes, as shown in Figs. S13a-c. The sum distribution of these three degenerate modes is shown in Fig. S13d.


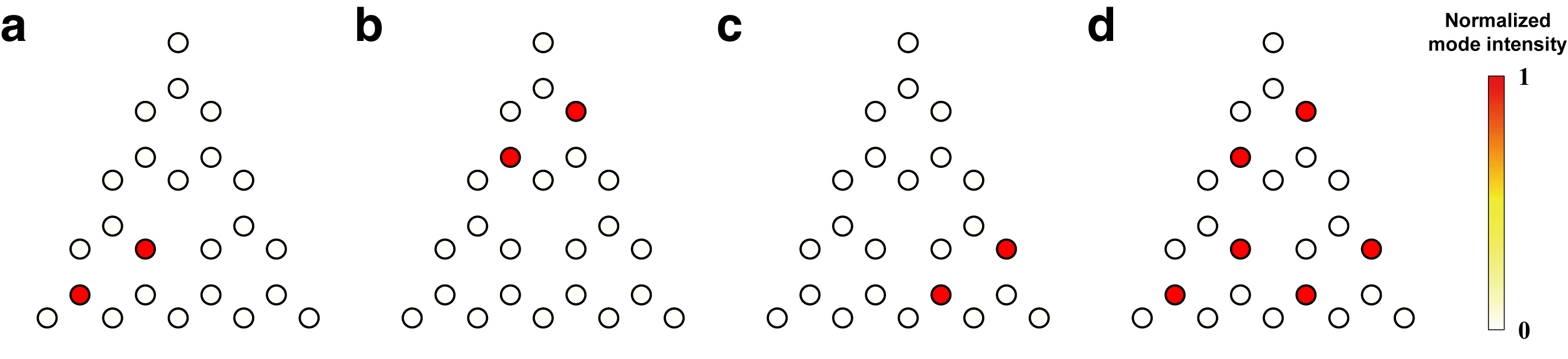


**Fig. S13 | Three inner edge modes IE_B_ of the fractal AFTI (a-c) and their sum distribution (d) in the Sierpinski gasket lattice**.

1. **Quantum source and circuit design**

The quantum two-photon source at 808 nm with V polarization is generated by pumping a Type-I BBO crystal using 404 nm CW laser via Type-I SPDC process. Before injecting into the fractal photonic lattice, we conduct the two-photon quantum interference of the single-photon pairs on a balanced (1:1) fiber beam splitter. By tuning the delay between the two-way single photons, we acquire the quantum interference curve, as shown in Fig. S14. The measured interference visibility is *V*_FBS_=96.8±0.3%, which suggests the good indistinguishability of the input single photons.

**
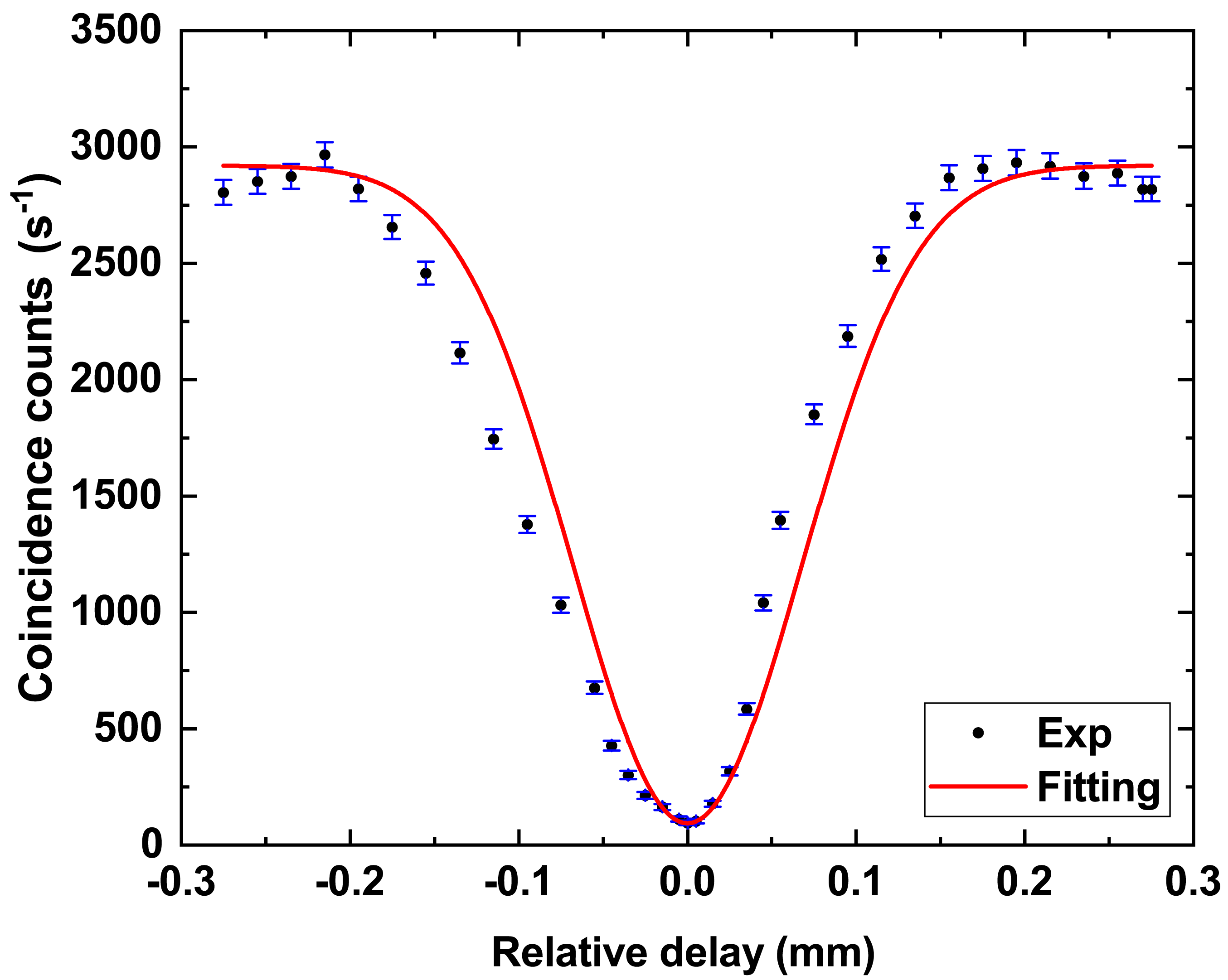
**

**Fig. S14 | The two-photon quantum interference curve of the quantum source on a balanced (1:1) fiber beam splitter.** The interference visibility is *V*_FBS_=96.8±0.3%. All error bars are calculated by assuming Poisson statistics.

Single photons undergo quantum interference on a balanced directional coupler (DC) can generate biphoton NOON state due to the bunching effect and further propagate as the outer/inner edge entangled state in the subsequent lattice. As shown in the cascading method in Fig. S15, the first photonic lattice is used to generate a pair of indistinguishable single-photon chiral edge states, and the followed DC (1:1) is for two-photon quantum interference to generate biphoton NOON state, $\left(|20\rangle+|02\rangle\right)/\sqrt{2}$. After interference, the generated NOON state is injected into the second photonic lattice to be transformed as the outer/inner edge entangled state via spatial or chiral encoding, $\left(|oo\rangle+|ii\rangle\right)/\sqrt{2}$or where $|o\rangle$($|i\rangle$) represents outer edge (inner edge) state and () represents the anticlockwise (clockwise) edge state. The quantum topological edge transport can also protect the generated outer/inner edge entangled state against imperfections and enable its complete transfer in the lattice.

**
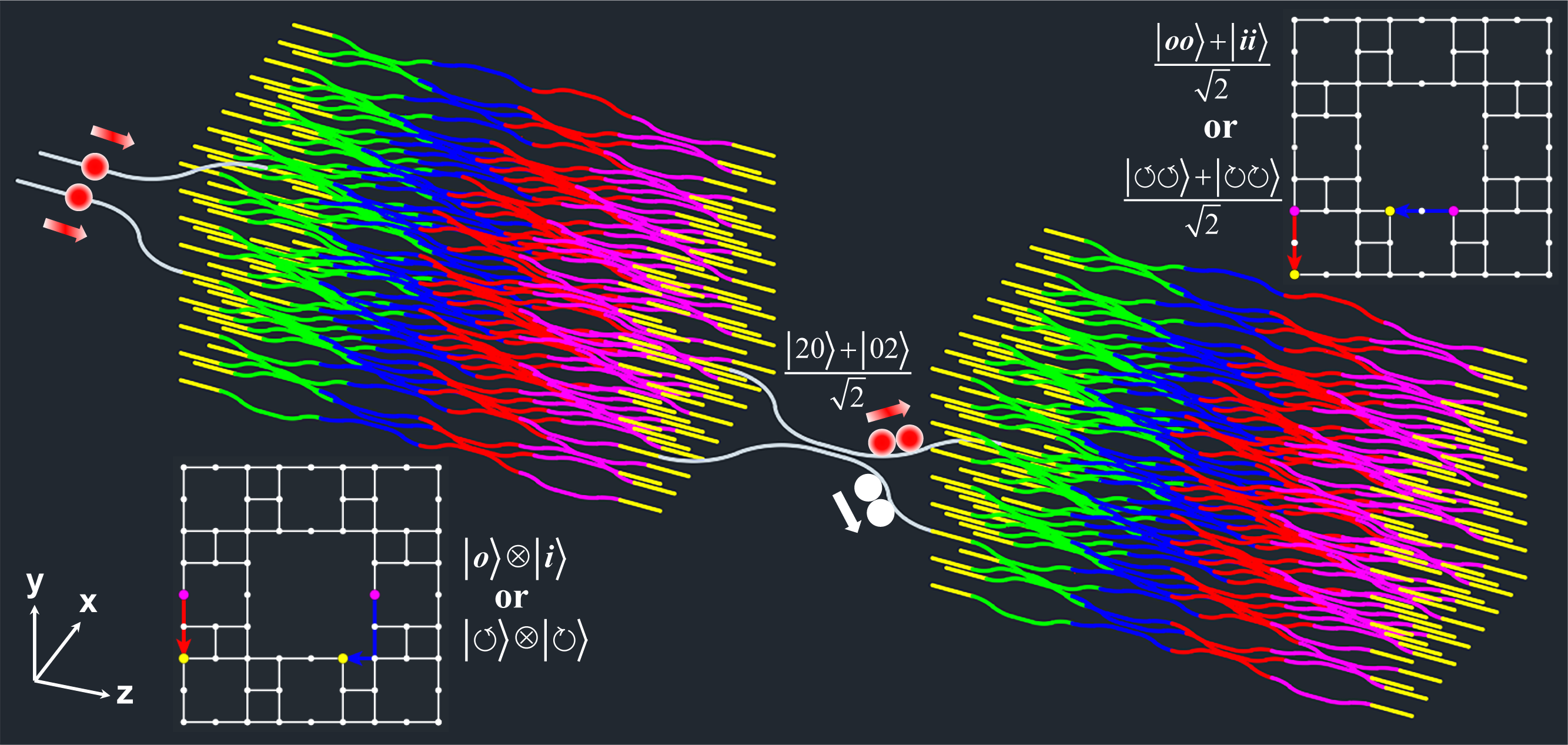
**

**Fig. S15 | Sketch of the cascading method to generate and propagate outer/inner chiral edge entangled state in fractal photonic AFTIs.** Pink (yellow) dots represents input (output) sites. The red and blue arrows represent the unidirectional transport of outer edge states and inner edge IE_A_ states, respectively.\

1. **Parameters for fabricating fractal photonic lattices**

In our experiment, we use a microscope objective with NA of 0.5 (20×) and fabricate waveguides from 30 μm to 270 μm below the surface. By optimally selecting the pulse energy of 470 nJ and the scanning speed of 20 mm/s, all the fabricated waveguides work in single mode with similar performances. The extra power compensation by the acoustic optical modulator and the correction of spherical aberration by the spatial light modulator are not applied, because it is hard to synchronize due to the fast scanning speed. Although the cross sections of waveguides fabricated in different depths indeed have slight variations, as shown in Fig. S16, they don’t dramatically influence experimental results.

Parameters for fabricating different horizontal and vertical directional couplers (DCs) to construct fractal photonic lattices (Sierpinski carpet) are shown in Table. S1 and Table. S2, respectively. For each DC, *L* is the interaction length, and *d* is the interaction distance. *T* represents the measured transmittance of DCs, which should be as close to 100% as possible.


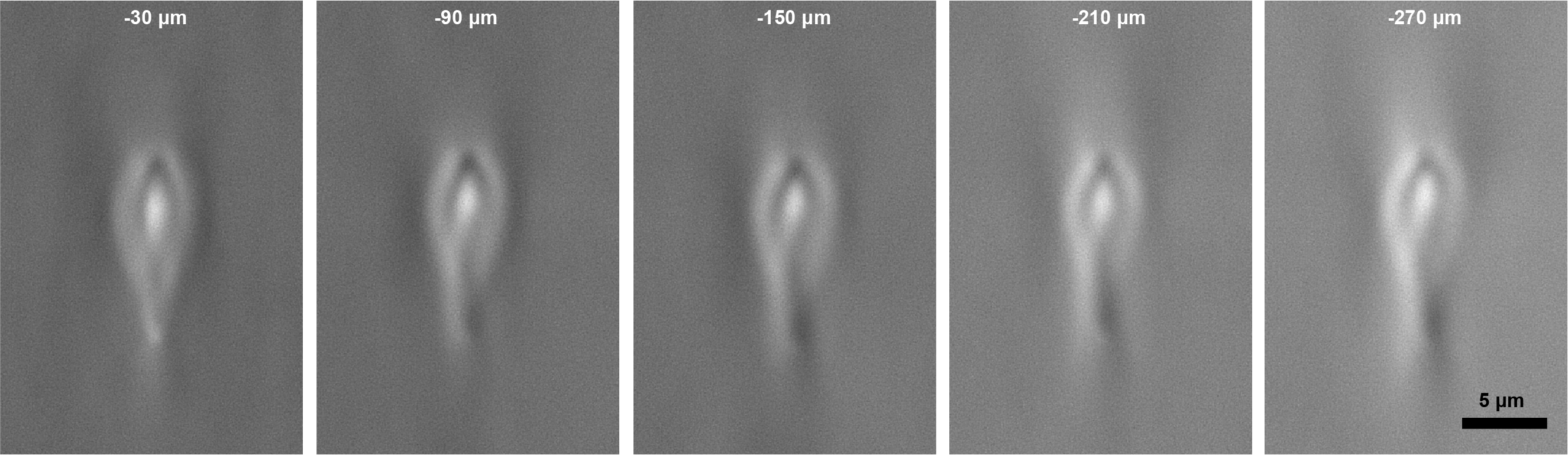


**Fig. S16 | Enlarged micrographs of waveguide cross sections at five different depths.**

**Table. S1 |** **Parameters for horizontal (XY) DCs with *d*=8 μm at 9 different layers**

| **Depth (μm)** | **30** | **60** | **90** | **120** | **150** | **180** | **210** | **240** | **270** |
| --- | --- | --- | --- | --- | --- | --- | --- | --- | --- |
| ***L* (mm)** | 1.10 | 1.30 | 1.20 | 1.20 | 1.10 | 1.40 | 1.50 | 1.70 | 1.30 |
| ***T* (%)** | 97.7 | 98.5 | 96.1 | 97.9 | 94.9 | 95.7 | 99.2 | 97.0 | 93.3 |

**Table. S2 |** **Parameters for vertical (XZ) DCs with *d*=10 μm at 8 different gaps**

| **Depth (μm)** | **(30,60)** | **(60,90)** | **(90,120)** | **(120,150)** | **(150,180)** | **(180,210)** | **(210,240)** | **(240,270)** |
| --- | --- | --- | --- | --- | --- | --- | --- | --- |
| ***L* (mm)** | 1.80 | 2.20 | 2.20 | 2.20 | 2.60 | 1.90 | 2.40 | 2.00 |
| ***T* (%)** | 96.8 | 97.8 | 98.8 | 99.2 | 98.5 | 88.0 | 98.2 | 92.0 |

1. **Particularities of the fractal AFTI lattice**

Thanks to the self-similarity, the fractal AFTI lattice has the advantages of the well-defined symmetry, geometrical scalability, and internal connectivity, which can’t be realized by the AFTI lattice with randomly missed bulk sites though it can also preserve outer edge modes and support inner edge modes associated with the inner boundaries of lattice holes because the path taken by wave packets in the AFTI is explicitly defined by the discrete driving protocol.

Fractal structures are made of building blocks displaying self-similarity of multiple scale, so the fractal lattice has a well-defined symmetry that includes the specific holes at different generations. However, the interior of the lattice with randomly missed bulk sites become more and more fragmented with the increasing lattice size, more easily breaking the symmetry.

Fractal structures have a good geometrical scalability in producing higher generations through its iteration procedure, so the fractal dimension of the lattice with dual Sierpinski carpet structure can maintain 1.89D at every generation. Apart from the lattice structure, the fractal dimension of chiral edge states ($D_{\text{e}}$) is also 1.89D when the number of generation (n) tends to infinity. The total number of lattice sites occupied by edge states including outer edge states and inner edge states is $N_{\text{e}}$, and the number of lattice sites on one length side of the fractal lattice is $N_{\text{l}}$. Therefore, the fractal dimension of edge states is:

$$D_{\text{e}}=\lim_{n\to\infty} \frac{\ln(N_{\text{e}})}{\ln(N_{\text{l}})}=\lim_{n\to\infty} \frac{\ln[(2\times3^{n}-1)+(2\times3^{n-1}+1)+\sum_{i=1}^{n-1} 8^{n-i}\times(2\times3^{i-1}+1)]}{\ln(3^{n})}=\frac{\ln8}{\ln3}\approx1.89$$

However, the random lattice without well-defined removal rules can’t guarantee this feature for its lattice and its edge states, so it is unscalable in geometry. For the practical application in multistate topological quantum system or large-scale linear optical quantum computation, the scalability of the system is very important. Fractal lattices can provide a workable method for the scalable and controllable mode encoding with the increase of generation, but lattices with randomly missed sites can’t.

Although lacking 17 bulk sites, the fractal AFTI lattice retains a systematically connected internal structure by virtue of the hierarchy of square holes. The quasi-energy spectrum of the fractal lattice has self-similarity and shows the existence of degenerate inner edge modes IE_B_, as shown in the Fig. 2a in the text. When we randomly remove 17 bulk sites from the 9×9 square lattice to generate the random lattice in Fig. S17a, the fragmented lattice structures destroy the internal connectivity, and even produce some isolated sites (the blue site in Fig. S17a). What’s more, the self-similarity of quasi energy spectrum disappears, and the number of degenerate edge modes is reduced, as shown in Fig. S17b. By analyzing some edge modes in this random lattice with a small deviation of coupling strength *δ*(Λ)/Λ_0_=0.1, the robustness of some edge modes against the small deviation is found weaker than that of others, as shown in Fig. S18. Therefore, the overall robustness of chiral edge modes in the fractal AFTI lattice is stronger than that in the AFTI lattice with randomly missed bulk sites.


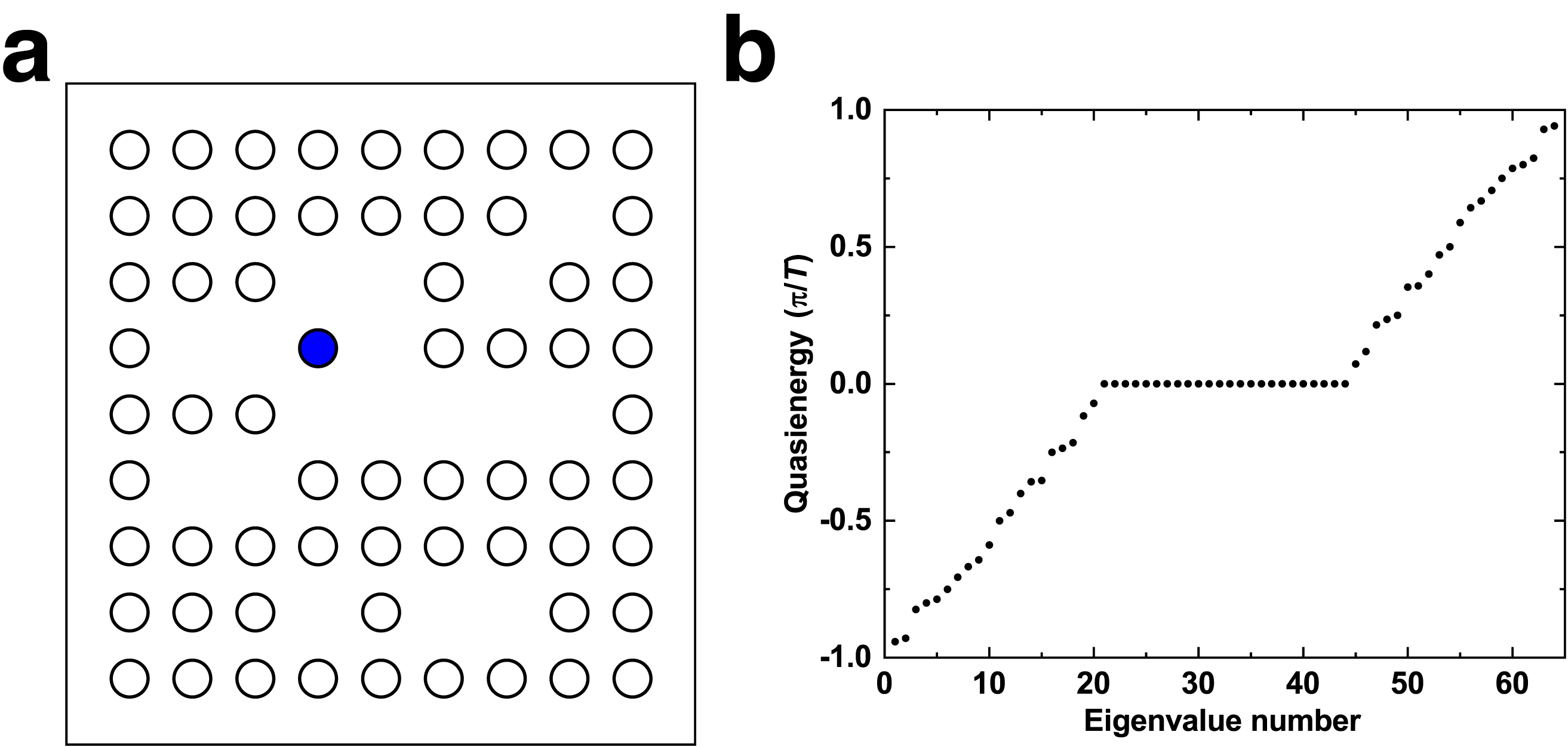


**Fig. S17 | A lattice with 17 randomly missed bulk sites.** **a,** The lattice structure is fragmented and the isolated site marked in blue appears. **b,** Quasi energy spectrum of this lattice shows no self-similarity, and no degenerate inner edge mode, and the gaps of quasi energy for some eigenstates are small.


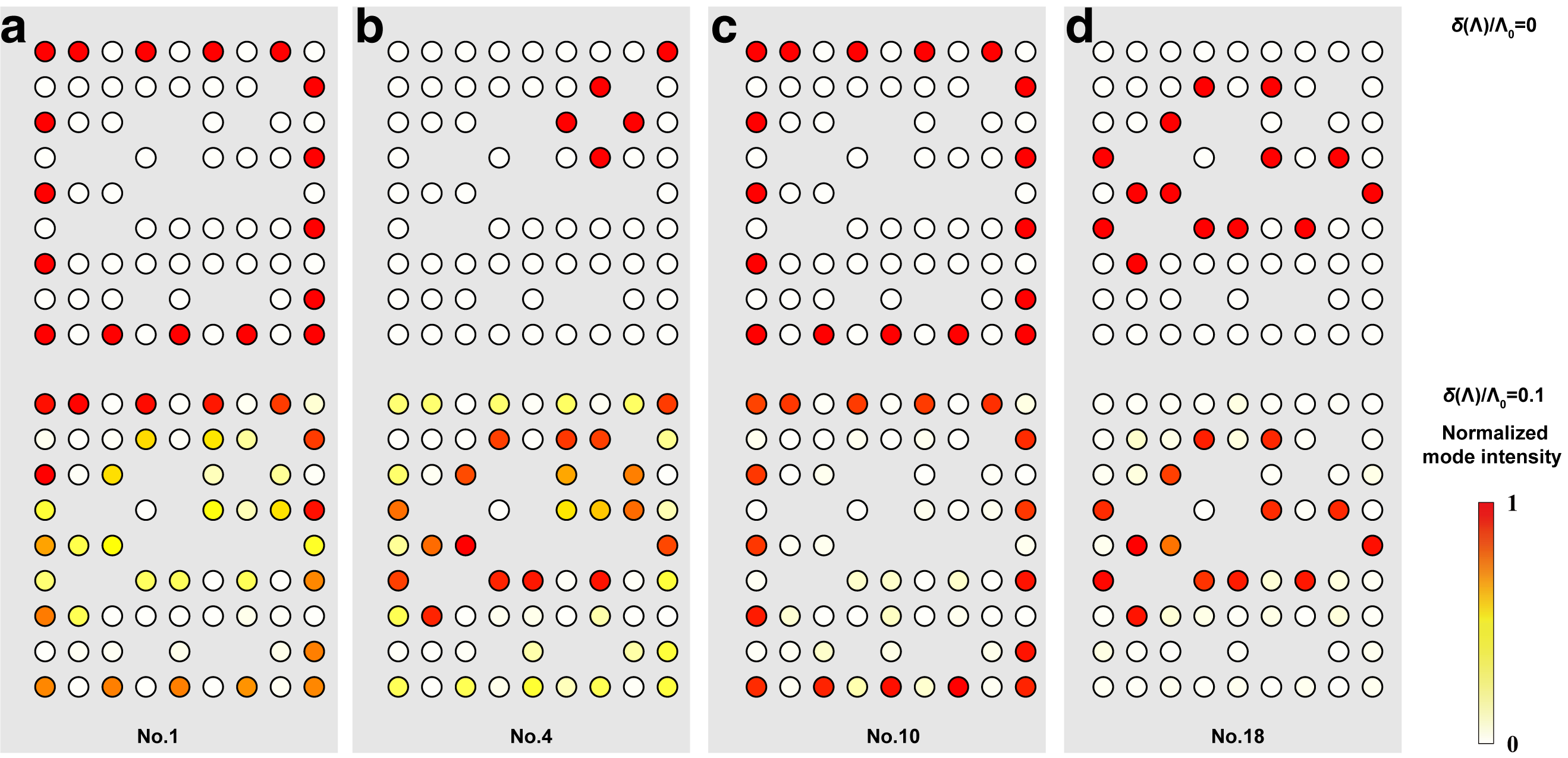


**Fig. S18 | Comparison of distributions of the field intensity of some edge modes in one 9x9 lattice with 17 randomly missed bulk sites when deviations of coupling strength *δ*(Λ)/Λ_0_ are 0 and 0.1, respectively.** With a small deviation, modes in **a** and **b** are mixed with other modes easily, but modes in **c** and **d** remain nearly unchanged. The robustness of modes in **a** and **b** are weaker than that of modes in **c** and **d**.

1. **Topological protection on the quantum correlation**

In this work, what we realize is to generate multiple single-photon chiral edge states with topologically protected quantum correlation and use the HOM interference experiment to prove they are highly indistinguishable. The high-visibility quantum interference relies on the indistinguishability of the two input photons instead of topological modes. Topological chiral edge modes protect the quantum correlation, though not the indistinguishability of single photons, making the measurement of HOM interference easy.

The lattice is equivalent to an environment with noise and loss, which make the quantum state fragile during the transport. Usually, the coincidence counts of correlated photon pairs will drop observably after propagation in the lattice due to various losses. If the coincidence counts further drop due to the reduction of two-photon correlation function, it is difficult to measure the HOM interference visibility with a low Signal-to-Noise Ratio (SNR). The HOM interference visibility is$V=\frac{C_{\max}-C_{\min}}{C_{\max}}=1-\frac{C_{\min}}{C_{\max}}$, and its uncertainty is$\varepsilon_{V}=\frac{C_{\min}}{C_{\max}}\sqrt{\frac{1}{C_{\max}}+\frac{1}{C_{\min}}}$. If the measured maximum coincidence count *C*_max_ is very low and the minimum coincidence count *C*_min_ is not easy to reduce, the measured interference visibility *V* is lower than the actual value and its uncertainty$\varepsilon_{V}$is high. To increase the coincidence counts, we can increase the pump power to inject more single photons, but that will induce the multi-photon term to further reduce the interference visibility. What’s more, the residual pump light will accidently coincide with the single photons in the detectors to increase the detection noise. Lengthening the integral time seems an alternative method, but the long time may introduce some unstable factors, such as the shift of the position with a relative delay of zero.

The topological chiral edge modes in the fractal AFTI lattice topologically protect quantum state transport of single photons to preserve the quantum correlation at a high level as that at the injection. Therefore, the coincidence counts for detection is so high that the HOM interference experiment is easy to conduct, and the measured interference visibility is close to the real value, which is beneficial to the application in optical quantum information processing.

The quantum correlation of two single photons propagating in the lattice can be described by the two-photon correlation function $\Gamma_{m,n}(z)=\left|U_{m,k}(z)U_{n,l}(z)+U_{m,l}(z)U_{n,k}(z)\right|^{2}$. As an example, when launching two indistinguishable photons into the lattice sites *k*=6 and *l*=22, the correlation function$\Gamma_{m,n}$after the evolutional distance of 2z_0_ (4z_0_) should be$\Gamma_{41,51}$($\Gamma_{48,32}$). As shown in Fig. S19, with the increase of deviation from Λ_0_=π/2 and evolutional distance, the correlation function of two photons output from waveguides for detection decreases dramatically. When the deviation is small, the quantum correlation is topologically protected, so it can maintain at a high level and decay slowly. But when the deviation is large enough, without topological protection, the quantum correlation decays very quickly. Even in a short evolution distance, almost no photons can be detected from the output sites.

Based on perfect hopping protocol, all photons of edge states are just distributed at their corresponding lattice sites and the quantum correlation has no change, as shown in Figs. 20a and 20d. However, when there exists the deviation of coupling strength, part of photons will escape from the designed trajectory and the quantum correlation will be reduced. The fractal AFTI remains topological when Λ*_i_* deviates from π/2 in a proper range, so main photons of edge states are still distributed at their corresponding lattice sites, as shown in Fig. S20b, and topological chiral edge modes can preserve two-photon correlation functions at a high level, as shown in Fig. S20e. But when the deviations become large enough, main photons of edge states escape from their corresponding lattice sites (Fig. S20c) and the quantum correlation is reduced obviously (Fig. S20f). What’s more, there exist some other points whose quantum correlations are close to that of the target points in the correlation map (Fig. S20f), which may result in the disturbance in the measurement.


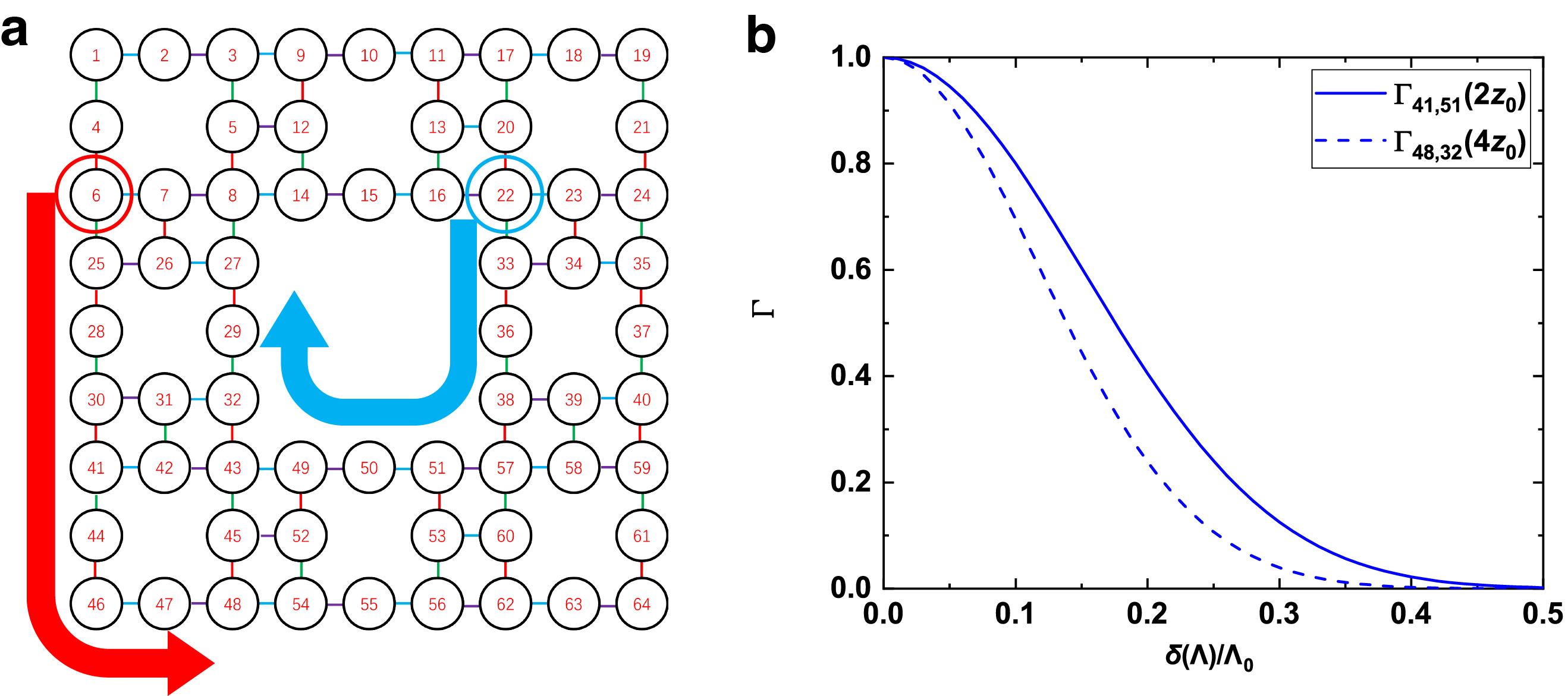


**Figure. S19 |** **Calculations of the two-photon correlation function**. **a,** When one single photon is injected into channel 6 (22), the fractal lattice will generate an anticlockwise (clockwise) outer (inner) edge state. **b,** The variation of the two-photon correlation function Γ_41,51_ (Γ_48,32_) after 2z_0_ (4z_0_) with the deviation of coupling strength *δ*(Λ)/Λ_0_.


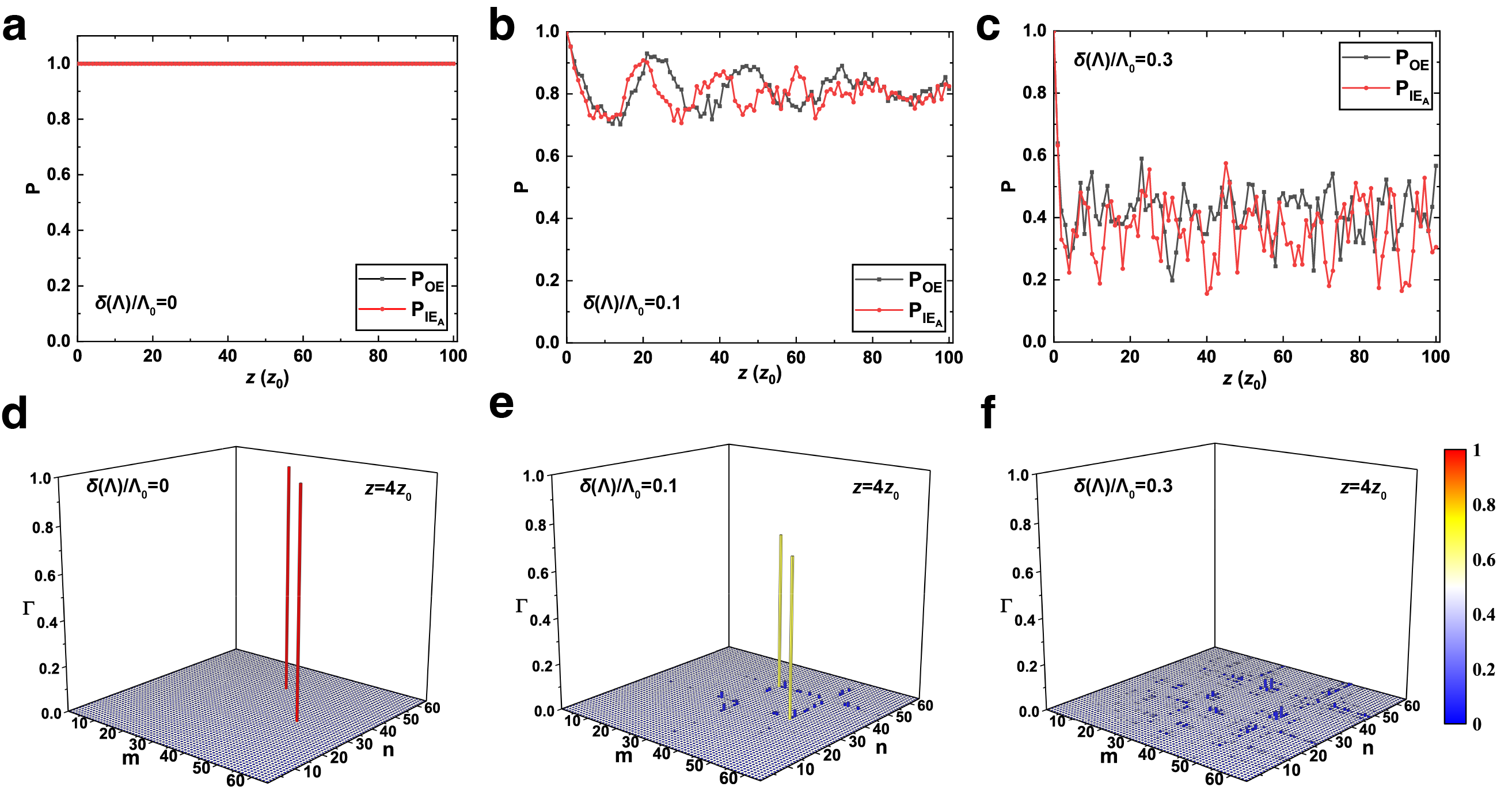


**Figure. S20 | Topological protection on mode distribution and quantum correlation.** The variation of the total field intensity of outer edge states (OE) and inner edge states IE_A_ with the propagation distance z when single photons are solely injected into lattice sites 6 and 22, respectively. The three lattices have different deviations of coupling strength. **a,** *δ*(Λ)/Λ_0_=0; **b,** *δ*(Λ)/Λ_0_=0.1; **c,** *δ*(Λ)/Λ_0_=0.3. Two-photon correlation function Γ_m,n_ after the propagation distance of 4z_0_ when a pair of single photons are injected into lattice sites (6, 22). The three lattices have different deviations of coupling strength. **d,** *δ*(Λ)/Λ_0_=0; **e,** *δ*(Λ)/Λ_0_=0.1; **f,** *δ*(Λ)/Λ_0_=0.3.

1. **Reference**

1. Rudner, M. S. *et al*. Anomalous edge states and the bulk-edge correspondence for periodically driven two-dimensional systems. *Physical Review X* **3**, 031005 (2013).

2. Maczewsky, L. J. *et al*. Observation of photonic anomalous Floquet topological insulators. *Nature Communications* **8**, 13756 (2017).

3. Mukherjee, S. *et al*. Experimental observation of anomalous topological edge modes in a slowly driven photonic lattice. *Nature Communications* **8**, 13918 (2017).

4. Mitchell, N. P., et al. Amorphous topological insulators constructed from random point sets. *Nature Physics* **14,** 380-385 (2018).

5. Yang, Z. J. *et al*. Photonic Floquet topological insulators in a fractal lattice. *Light: Science & Applications* **9**, 128 (2020).

6. Biesenthal, T. *et al*. Fractal photonic topological insulators. *Science* **376**, 1114-1119 (2022).

7. Bandres, M. A., Rechtsman, M. C. & Segev, M. Topological photonic quasicrystals: fractal topological spectrum and protected transport. *Physical Review X* **6**, 011016 (2016).

8. Mondragon-Shem, I., et al. Topological Criticality in the Chiral-Symmetric AIII Class at Strong Disorder. *Physical Review Letters* **113,** 046802 (2014).

9. Song, J. & Prodan, E. AIII and BDI topological systems at strong disorder. *Physical Review B* **89,** 224203 (2014).

10. Meier, E. J., et al. Observation of the topological Anderson insulator in disordered atomic wires. *Science* **362,** 929-933 (2018).

11. Lin, L., Ke, Y. G. & Lee, C. H. Real-space representation of the winding number for a one-dimensional chiral-symmetric topological insulator. *Physical Review B* **103,** 224208 (2021).
